# Clinical utility of combining the C-reactive protein-to-albumin ratio and systemic immune-inflammation index for prognostic assessment in metastatic renal cell carcinoma

**DOI:** 10.1371/journal.pone.0345724

**Published:** 2026-10-07

**Authors:** Nobuhito Muramoto, Masayoshi Nagata, Hisashi Hirano, Tomoya Shirakawa, Toshiyuki China, Haruna Kawano, Shuji Isotani, Hisamitsu Ide, Hiroaki Honda, Shigeo Horie

**Affiliations:** 1 Department of Urology, Juntendo University, Graduate School of Medicine, Tokyo, Japan; 2 Human Disease Models, Institute of Laboratory Animals, Tokyo Women’s Medical University, Tokyo, Japan; 3 Data Science and Informatics for Genetic Disorders, Juntendo University, Graduate School of Medicine, Tokyo, Japan; 4 Innovative Longevity, Juntendo University, Graduate School of Medicine, Tokyo, Japan; Iuliu Hațieganu University of Medicine and Pharmacy: Universitatea de Medicina si Farmacie Iuliu Hatieganu, ROMANIA

## Abstract

**Background:**

Inflammation-based biomarkers may refine prognostic stratification in patients with metastatic renal cell carcinoma (mRCC). This study evaluated the prognostic value of the pretreatment C-reactive protein-to-albumin ratio (CAR) and systemic immune-inflammation index (SII) in patients with mRCC receiving first-line systemic therapy.

**Methods:**

We retrospectively analyzed 117 patients who received first-line systemic therapy between January 2001 and April 2022. Pretreatment CAR and SII were evaluated as readily available blood-based inflammatory biomarkers. Overall survival (OS) was defined as the primary outcome, and progression-free survival (PFS) was defined as the secondary outcome. Exploratory cutoffs were CAR 0.0731 and SII 533.81.

**Results:**

High CAR and high SII were independently associated with shorter OS after adjustment for IMDC risk classification and nephrectomy status. The adjusted HRs were 2.36 for high CAR (95% CI, 1.30–4.27; *p* = 0.005) and 2.58 for high SII (95% CI, 1.42–4.69; *p* = 0.002). Patients with concurrent elevation of both CAR and SII had the shortest OS compared with those with neither marker elevated (HR, 6.39; 95% CI, 2.76–14.77; *p* < 0.001). Adding CAR and SII to International Metastatic Renal Cell Carcinoma Database Consortium (IMDC) improved the optimism-corrected C-index for OS from 0.641 to 0.733.

**Conclusions:**

Pretreatment CAR and SII were associated with OS in patients with mRCC receiving first-line systemic therapy. These readily available biomarkers may help refine OS risk assessment when used alongside established clinical factors, but external validation is required before clinical implementation.

## Introduction

Kidney cancer is a common malignancy worldwide, accounting for approximately 2.2% of all newly diagnosed cancers [1,2]. In 2022, approximately 435,000 new cases of kidney cancer and 156,000 kidney cancer-related deaths were reported worldwide [1]. Although incidence rates vary geographically, Asia contributes substantially to the global number of kidney cancer cases because of its large population, supporting the importance of evaluating prognostic markers in Asian clinical cohorts [1,3]. When diagnosed at a localized stage, renal cell carcinoma (RCC) can often be managed with curative-intent local therapy, including partial or radical nephrectomy, and generally has a favorable prognosis, with a 5-year relative survival rate of approximately 93% [2].

However, some patients present with metastatic disease at diagnosis, whereas others develop recurrence or metastasis after treatment for localized disease. Although the treatment landscape of metastatic renal cell carcinoma (mRCC) has improved substantially with the introduction of targeted therapies and immune checkpoint inhibitor-based regimens, prognosis remains heterogeneous and limited in the metastatic setting [4–7]. Therefore, simple and clinically accessible biomarkers that can refine prognostic assessment at the initiation of first-line systemic therapy are needed [8].

The International Metastatic Renal Cell Carcinoma Database Consortium (IMDC) risk classification is widely used for prognostic stratification in patients with mRCC [8,9]. However, prognosis can still vary among patients within the same risk category, and additional biomarkers based on routinely available clinical data may help refine risk assessment. Systemic therapy for mRCC has evolved from cytokine-based therapy to targeted therapies and, more recently, immune checkpoint inhibitor-based regimens [4–7,10,11]. In this changing therapeutic landscape, accessible biomarkers that can complement established clinical risk models may be useful for individualized prognostic assessment.

Systemic inflammation is closely linked to cancer progression, metastasis, and survival. In RCC, inflammatory pathways and the host inflammatory response have been implicated in tumor progression and clinical prognosis [12,13]. Accordingly, several blood-based inflammatory and nutrition-related indices have been investigated in relation to survival in patients with mRCC [14–17]. These include the C-reactive protein-to-albumin ratio (CAR), neutrophil-to-lymphocyte ratio (NLR), platelet-to-lymphocyte ratio (PLR), monocyte-to-lymphocyte ratio (MLR), systemic immune-inflammation index (SII), modified Glasgow Prognostic Score (mGPS), prognostic nutritional index (PNI), and geriatric nutritional risk index (GNRI) [14,15,18–20]. These indices capture different aspects of systemic inflammation, immune status, and nutritional reserve. The NLR reflects neutrophil-dominant systemic inflammation relative to lymphocyte-mediated immune status, whereas the PLR and MLR incorporate platelet- or monocyte-associated inflammatory activity in relation to lymphocyte counts [14,18]. The mGPS combines C-reactive protein (CRP) and albumin and represents an inflammation-based prognostic score [15]. The PNI incorporates serum albumin and lymphocyte count as a nutrition- and immune-related index, whereas the GNRI incorporates serum albumin and the ratio of body weight to ideal body weight as a marker of nutritional risk [19,20]. Among these markers, CAR and SII are particularly attractive because they can be calculated from routine pretreatment laboratory data and reflect complementary aspects of the host inflammatory response. As a composite biomarker, CAR integrates CRP and albumin, thereby capturing systemic inflammation and nutritional status [14,16], whereas SII incorporates neutrophil, platelet, and lymphocyte counts and reflects the balance between inflammation, thrombocytosis, and immune status [17]. Previous studies have reported associations between CAR or SII and survival in patients with mRCC [14,16,17]. However, reported cutoff values vary across studies, and the incremental prognostic value of combining CAR and SII with established clinical factors has not been fully clarified. In particular, limited data are available regarding whether the combined use of CAR and SII provides additional prognostic information when considered together with established clinical factors, and whether such information is supported by model performance measures including discrimination, prediction error, calibration, and decision curve analysis.

In this study, we retrospectively evaluated the prognostic value of CAR and SII in patients with mRCC receiving first-line systemic therapy. We assessed their associations with overall survival (OS) and progression-free survival (PFS), examined their combined prognostic value, and evaluated the incremental prognostic performance of adding CAR and SII to established clinical factors.

## Materials and methods

### Patients and data collection

We retrospectively reviewed medical records of patients with mRCC who received systemic therapy between January 2001 and April 2022 at the Department of Urology, Juntendo University Hospital, Tokyo, Japan. The study was approved by the ethics committee of Juntendo University on September 28, 2023 (E23-0275-H01). The requirement for individual informed consent was waived by the ethics committee, and an opt-out procedure was implemented in accordance with institutional policy. The data were accessed for research purposes in December 2023. The data were extracted from the electronic medical record system. The authors had access to information that could identify individual participants during data collection. All analyses were performed using de-identified data. Patients were excluded if pretreatment blood samples were not obtained prior to initiation of systemic therapy or if survival outcomes could not be confirmed from the medical records. Baseline laboratory values (complete blood count, albumin, CRP, calcium, and lactate dehydrogenase) were collected prior to treatment initiation. Inflammatory and nutritional indices were calculated as follows: NLR = N/L, PLR = P/L, and MLR = M/L; SII = neutrophil×platelet/lymphocyte using pretreatment counts. The PNI, GNRI, and mGPS were calculated using pretreatment laboratory data. PNI was calculated as follows: 10 × serum albumin concentration (g/dL) + 0.005 × total lymphocyte count (/mm³) [21]. GNRI was calculated as follows: 14.89 × serum albumin concentration (g/dL) + 41.7 × body weight/ideal body weight. When body weight exceeded ideal body weight, the body weight/ideal body weight ratio was set to 1 [22]. Ideal body weight was calculated as 22 × height² (m²). The mGPS was assigned as follows: patients with CRP ≤ 1.0 mg/dL were assigned a score of 0; patients with CRP > 1.0 mg/dL and serum albumin ≥ 3.5 g/dL were assigned a score of 1; and patients with CRP > 1.0 mg/dL and serum albumin < 3.5 g/dL were assigned a score of 2 [23]. No missing data were observed for the variables included in the analyses.

### Determination of cutoff values for inflammatory and nutritional markers

Time-dependent receiver operating characteristic (ROC) analyses for censored survival outcomes were performed to evaluate discrimination of inflammatory indices for OS. For graphical risk stratification, exploratory cutoffs for CAR, NLR, PLR, MLR, and SII were derived using the Youden index at the prespecified time horizon (5 years), and the stability of discrimination at 1 and 3 years was assessed as a sensitivity analysis. Because reported cutoff values for inflammatory indices in metastatic RCC vary across studies, the cutoff values used in the present study were considered exploratory and cohort-derived. In contrast, PNI and GNRI were dichotomized using previously reported cutoff values of 40 and 98, respectively, and these cutoffs were not derived from the present cohort [21,22]. High and low CAR were defined as CAR ≥ 0.0731 and CAR < 0.0731, respectively. High and low SII were defined as SII ≥ 533.81 and SII < 533.81, respectively.

### Statistical analysis

OS was defined as the primary outcome and PFS as the secondary outcome, and both were evaluated using the Kaplan–Meier method and log-rank tests. PFS was defined as the time from initiation of first-line systemic therapy to the first documented disease progression according to Response Evaluation Criteria in Solid Tumors version 1.1 criteria or death from any cause, whichever occurred first. OS was defined as the time from initiation of first-line systemic therapy to death from any cause or last follow-up. Because CAR and SII were measured immediately before the initiation of systemic therapy, this time origin was selected to align the timing of biomarker assessment with the start of survival follow-up.

Associations with PFS and OS were assessed using univariable and multivariable Cox proportional hazards models, reporting hazard ratios (HRs) with 95% confidence intervals (CIs). The main multivariable model was defined a priori for OS and included IMDC risk classification, nephrectomy status, high CAR, and high SII. The number of death events was 56. Because IMDC risk classification was entered as a three-level categorical variable, the main multivariable model included five model parameters: two parameters for IMDC risk classification, one for nephrectomy status, one for high CAR, and one for high SII. This corresponded to approximately 11 events per model parameter. Performance status was not added separately to the main model because it is included in the IMDC risk classification and was highly imbalanced in this cohort, with only two patients having Karnofsky performance status (KPS) < 80. Histology was not included in the primary model because histological subtype was unknown in 23 patients. Histological heterogeneity was instead assessed in a sensitivity analysis restricted to patients with confirmed clear-cell RCC. IMDC risk classification and nephrectomy status were selected as clinically relevant prognostic factors, whereas high CAR and high SII were included as the main biomarkers of interest. Variables were not selected on the basis of univariable *p* values. Because of the modest number of death events and the risk of overfitting, additional variables such as immune checkpoint inhibitor (ICI)-based first-line therapy and histological subtype were not included simultaneously in the main model but were assessed in sensitivity analyses. Additional exploratory multivariable models were constructed separately for other inflammatory and nutritional indices. Exploratory cutoffs for CAR, NLR, PLR, MLR, and SII were derived using time-dependent ROC analysis for OS at the prespecified 5-year time horizon and the Youden index. Because reported cutoff values for inflammatory indices in metastatic RCC vary across studies, the cutoffs used in the present study were considered exploratory and were not intended to define universal clinical thresholds. To reduce reliance on dichotomized cutoff values, CAR and SII were also analyzed as log-transformed standardized continuous variables, and HRs were reported per 1 standard deviation increase.

All statistical analyses were performed using EZR (Saitama Medical Center, Jichi Medical University, Saitama, Japan) or GraphPad Prism (version 10). All *p* values were two-sided and statistical significance was defined as *p* < 0.05. Incremental prognostic performance beyond IMDC was assessed using bootstrap internal validation (300 resamples) with optimism-corrected Harrell’s C-index. Time-dependent area under the curve (AUC) and Brier scores were used to summarize discrimination and prediction error at 1, 3, and 5 years. Decision curve analysis (DCA) was used to assess potential clinical net benefit where applicable.

## Results

### Patient characteristics

**Table 1** summarizes the baseline clinical characteristics of the 117 patients. The cohort included 83 men and 34 women, with a median age of 67 years. During a median follow-up of 72.4 months, 56 patients died. Nephrectomy was performed in 81 patients, whereas 36 patients did not undergo nephrectomy. Pathologically, 79 patients had clear cell RCC and 15 had non-clear cell RCC, while histology was unavailable for 23 patients because nephrectomy was not performed. First-line therapy consisted of mTOR inhibitors in 7 patients, TKIs in 86 patients, and immune checkpoint inhibitor-based therapy in 24 patients. According to IMDC risk classification, 6, 71, and 40 patients were categorized as favorable, intermediate, and poor risk, respectively. Baseline inflammatory and nutritional indices were calculated from pretreatment laboratory values, as defined in the Methods.

**Table 1:** Patient characteristics and univariable Cox regression analyses for overall survival.

Parameter	Total N = 117	Univariable HR for OS (95% CI)	*p* value
Sex (%)			
Male	83 (71)	0.69 (0.36–1.32)	0.273
Female	34 (29)	Reference	—
Age (median, IQR)	67 (61–76)		
Age > 65 years	71 (61)	1.61 (0.87–2.96)	0.122
Age ≤ 65 years	46 (39)	Reference	—
BMI (kg/m², median, IQR)	23.04 (20.46–25.36)		
BMI < 18 kg/m²	12 (10)	1.01 (0.55–1.84)	0.977
BMI ≥ 18 kg/m²	105 (90)	Reference	—
KPS (%)			
≥ 80	115 (98)	Reference	—
< 80	2 (2)	3.78 (0.91–15.72)	0.067
Nephrectomy (%)			
Yes	81 (69)	0.38 (0.22–0.68)	0.001
No	36 (31)	Reference	—
IMDC risk classification (%)			
Favorable	6 (5)	Reference	—
Intermediate	71 (60)	3.24 (0.44–24.03)	0.251
Poor	40 (35)	6.90 (0.93–51.28)	0.059
Histological subtype (%)			
Clear	79 (68)	Reference	—
Non-clear	15 (13)	1.80 (0.79–4.12)	0.164
Unknown	23 (19)	2.95 (1.62–5.37)	< 0.001
First-line therapy (%)			
mTORi	7 (6)		
TKI	86 (74)		
ICI-ICI	19 (16)		
ICI-TKI	5 (4)		
ICI-based first-line therapy	24 (21)	0.64 (0.25–1.62)	0.347
Non-ICI-based first-line therapy	93 (79)	Reference	—
Treatment lines (%)			
≤ 2	88 (75)	1.29 (0.72–2.34)	0.402
> 2	29 (25)	Reference	—
Neutrophil counts (median, IQR)	4571 (3240–5390)		
Lymphocyte counts (median, IQR)	1433 (968–1754)		
Platelet counts (median, IQR)	20.9 (17.3–24.5)		
Monocyte counts (median, IQR)	401.9 (266–498)		
Albumin concentration (g/dL, median, IQR)	3.74 (2.4–4.2)		
CRP level (mg/dL, median, IQR)	3.59 (0.27–3.76)		
Hb concentration (g/dL, median, IQR)	11.98 (10.5–12.95)		
Corrected calcium (mg/dL, median, IQR)	9.46 (9.1–9.7)		
NLR (median, IQR)	3.97 (2.196–4.207)		
NLR ≥ 3.732	41 (35)	2.89 (1.67–5.02)	< 0.001
NLR < 3.732	76 (65)	Reference	—
PLR (median, IQR)	1.548 (0.757–1.765)		
PLR ≥ 0.862	79 (68)	1.90 (1.05–3.43)	0.032
PLR < 0.862	38 (32)	Reference	—
MLR (median, IQR)	0.340 (0.174–0.386)		
MLR ≥ 0.525	19 (16)	2.49 (1.37–4.54)	0.002
MLR < 0.525	98 (84)	Reference	—
LMR (median, IQR)	3.784 (2.589–5.723)		
PNI (median, IQR)	38.152 (35.395–42.548)		
PNI < 40	62 (53)	2.24 (1.29–3.88)	0.003
PNI ≥ 40	55 (47)	Reference	—
GNRI (median, IQR)	97.93 (90.80–106.553)		
GNRI < 98	62 (53)	2.63 (1.52–4.56)	< 0.001
GNRI ≥ 98	55 (47)	Reference	—
mGPS (%)			
0	65 (56)	Reference	—
≥ 1	52 (44)	2.15 (1.27–3.66)	0.004
CAR (median, IQR)	0.075 (0.028–0.627)		
High CAR	61 (52)	2.70 (1.54–4.74)	< 0.001
Low CAR	56 (48)	Reference	—
SII (median, IQR)	456.32 (15.04–1022.01)		
High SII	55 (47)	2.80 (1.59–4.94)	< 0.001
Low SII	62 (53)	Reference	—

Values are presented as number (percentage) unless otherwise indicated. Continuous variables are presented as median with interquartile range. High and low CAR were defined as CAR ≥ 0.0731 and CAR < 0.0731, respectively. High and low SII were defined as SII ≥ 533.81 and SII < 533.81, respectively. IQR: interquartile range, KPS: Karnofsky Performance Status, IMDC: International Metastatic Renal Cell Carcinoma Database Consortium, BMI: body mass index, mTORi: mammalian target of rapamycin inhibitor TKI: tyrosine kinase inhibitor, ICI-ICI: combination of Immune checkpoint inhibitor, ICI-TKI: combination of Immune checkpoint inhibitor and tyrosine kinase inhibitor, CAR:C-reactive protein/albumin ratio, NLR: neutrophil-to-lymphocyte ratio, PLR: platelet-to-lymphocyte ratio, MLR: monocyte-to-lymphocyte ratio index PNI: prognostic nutritional index, GNRI: Geriatric Nutritional Risk Index, mGPS: modified Glasgow Prognostic Score, SII: systemic immune-inflammation index

### Cutoff determination and time-dependent ROC analyses

For graphical risk stratification, exploratory cutoffs were determined using the Youden index at the prespecified time horizon of five years. The optimal cutoffs were 0.0731 for CAR and 533.81 for SII, with corresponding 5-year AUCs of 0.656 (95% CI, 0.505–0.807) and 0.754 (95% CI, 0.614–0.895), respectively. Time-dependent ROC curves for CAR and SII are provided in Fig 1. Exploratory time-dependent ROC analyses for other inflammatory indices, including NLR, PLR, and MLR, are provided in S1 Fig. The AUC values shown in Fig 1 and S1 Fig. reflect time-dependent discrimination at each time point. In contrast, AUC values reported alongside the cutoff values correspond to the prespecified 5-year horizon used for cutoff determination.

**Fig 1:**
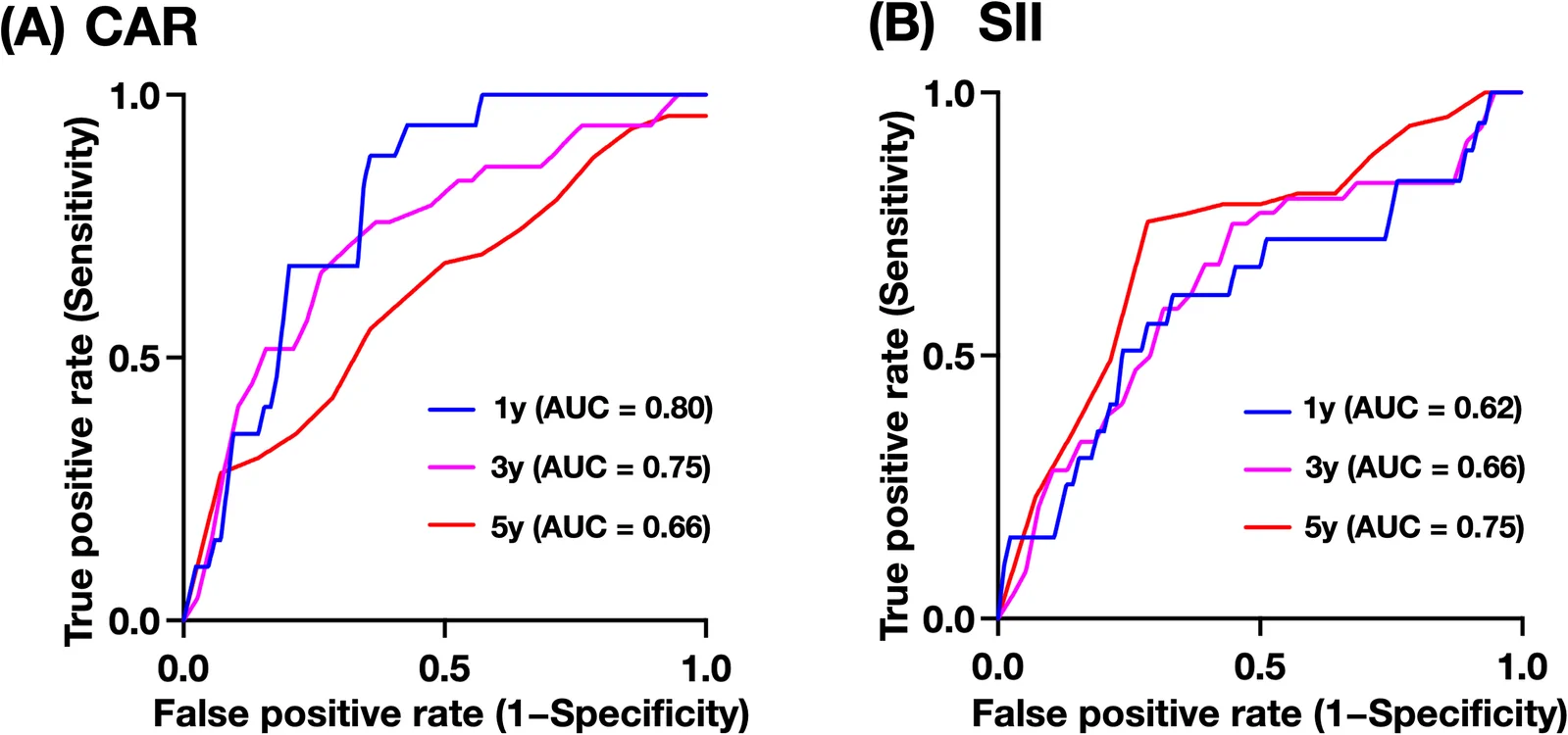
Time-dependent ROC curves for CAR and SII for OS. Panels show ROC curves at 1, 3, and 5 years. AUC values are reported in each panel. AUC: Area Under the Curve, CAR: C-reactive protein-to-albumin ratio, SII: systemic immune-inflammation index.

### Kaplan-Meier survival stratification by CAR and SII

Kaplan-Meier curves stratified by CAR and SII are shown in Fig 2. Patients with high CAR had significantly shorter progression free survival (PFS) than those with low CAR (HR, 2.47; 95% CI, 1.49–4.10; *p* = 0.001) (Fig 2(A)). Patients with high SII also had significantly shorter PFS than those with low SII (HR, 2.68; 95% CI, 1.58–4.54; *p* < 0.001) (Fig 2(B)). Similar associations were observed for OS. High CAR was associated with shorter OS (HR, 2.25; 95% CI, 1.35–3.73; *p* = 0.001) (Fig 2(C)). Patients with high SII had significantly shorter OS than those with low SII (HR, 2.75; 95% CI, 1.62–4.66; *p* < 0.001) (Fig 2(D)). To evaluate the combined effect of CAR and SII, patients were further stratified into three groups. These groups were low CAR and low SII, either high CAR or high SII, and both high CAR and high SII. The corresponding Kaplan-Meier curves are shown in Fig 3. Significant differences in PFS and OS were observed among the three groups by the log-rank test (PFS, *p* < 0.001; OS, *p* < 0.001). Both PFS and OS worsened stepwise across these groups, with the shortest PFS and OS observed in patients with high CAR/high SII. Compared with patients with low CAR/low SII, patients with high CAR/high SII had significantly shorter PFS (HR, 4.01; 95% CI, 1.98–8.13; *p* < 0.001) and OS (HR, 6.39; 95% CI, 2.76–14.77; *p* < 0.001).

**Fig 2:**
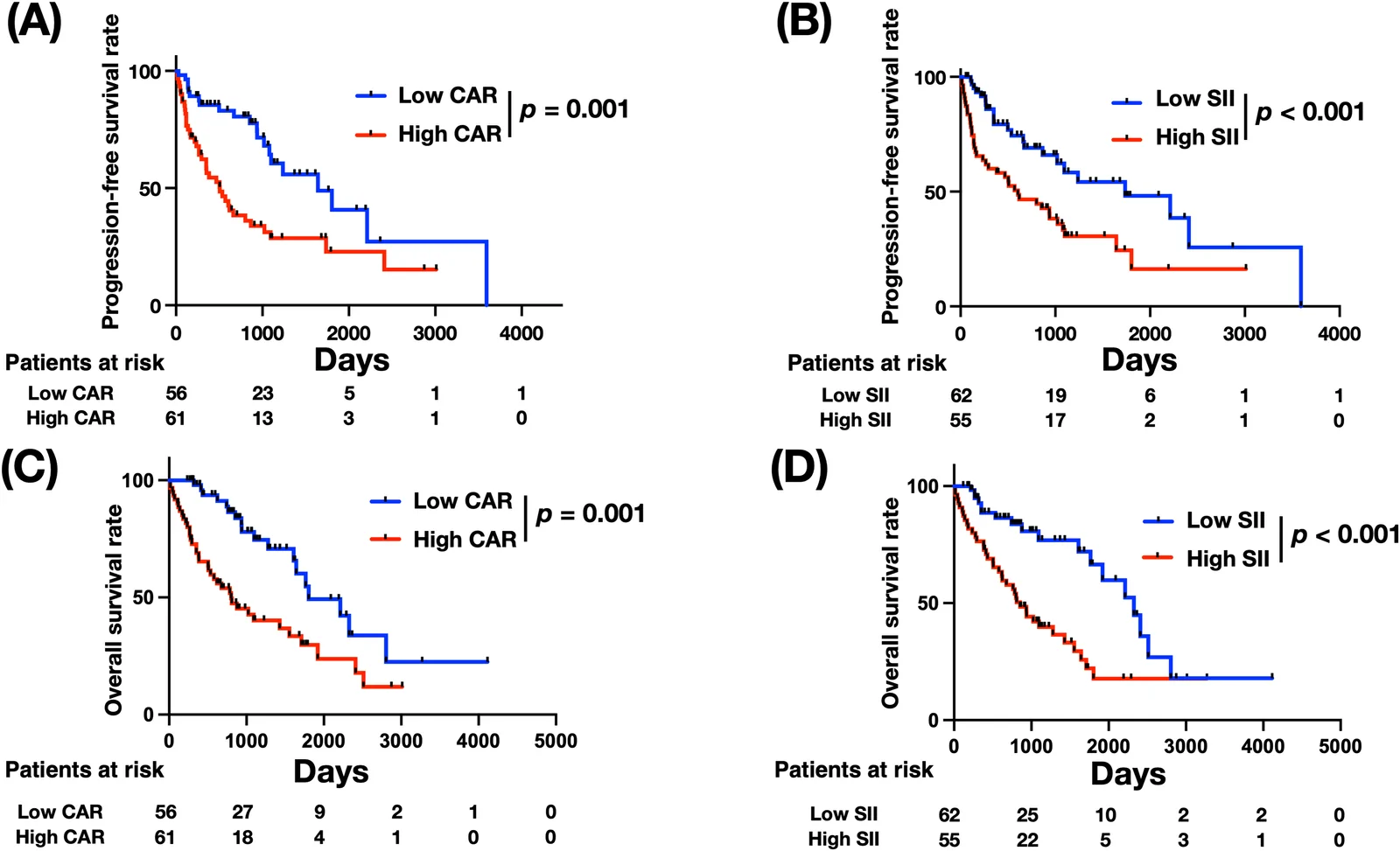
Kaplan–Meier curves for PFS and OS stratified by CAR and SII cutoffs. Panels show (A) PFS by CAR, (B) PFS by SII, (C) OS by CAR, and (D) OS by SII. High and low CAR were defined as CAR ≥ 0.0731 and CAR < 0.0731, respectively. High and low SII were defined as SII ≥ 533.81 and SII < 533.81, respectively. CAR: C-reactive protein-to-albumin ratio, SII: systemic immune-inflammation index, PFS: Progression Free Survival, OS: Overall Survival, Cutoffs: CAR 0.0731; SII 533.81.

**Fig 3:**
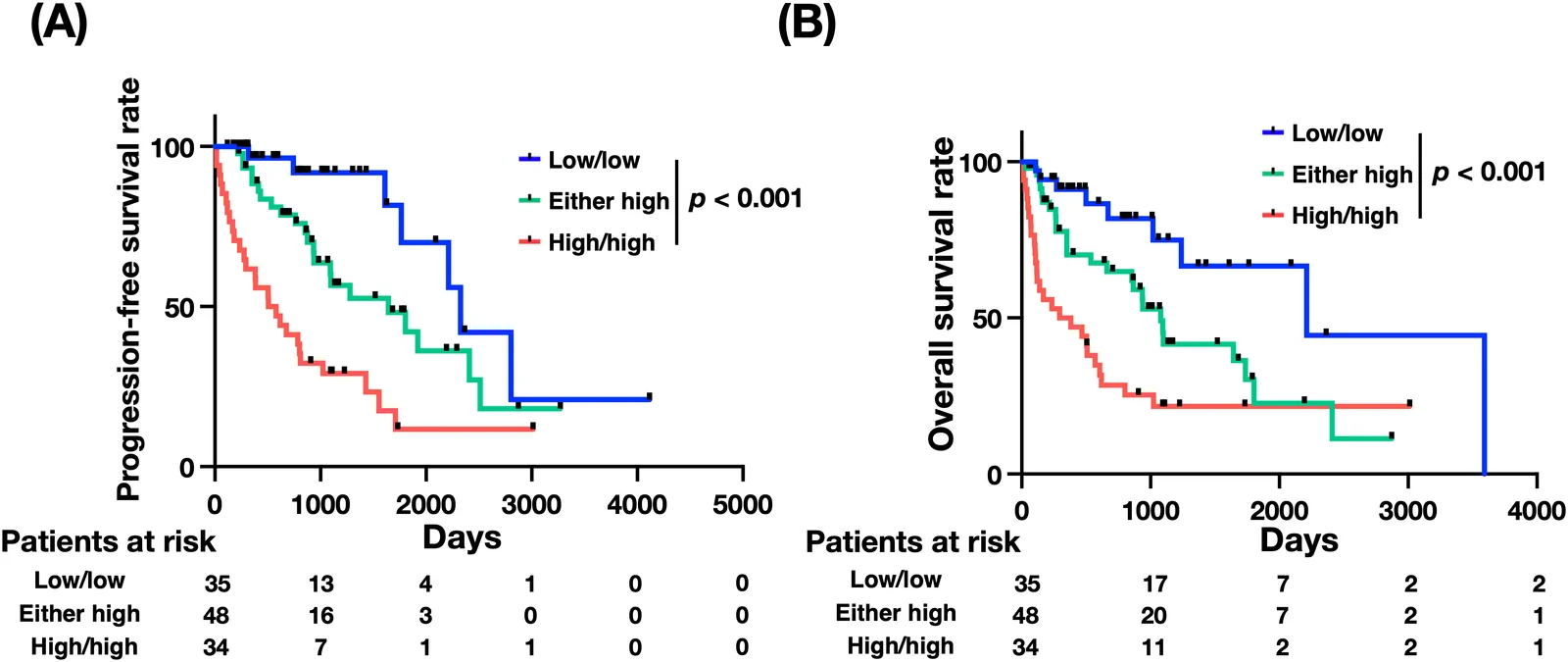
Kaplan–Meier curves stratified by combined CAR/SII groups. Patients were stratified into three groups: Low/low, Either high, and High/high. Low/low was defined as low CAR and low SII; Either high was defined as either high CAR or high SII; and High/high was defined as high CAR and high SII. Panels show (A) PFS and (B) OS. High and low CAR were defined as CAR ≥ 0.0731 and CAR < 0.0731, respectively. High and low SII were defined as SII ≥ 533.81 and SII < 533.81, respectively. CAR: C-reactive protein-to-albumin ratio, SII: systemic immune-inflammation index, PFS: Progression Free Survival, OS: Overall Survival.

### Cox regression analyses for overall survival

The main multivariable Cox model was defined a priori and included IMDC risk classification, nephrectomy status, high CAR, and high SII to reduce the risk of overfitting (**Table 2**). In univariable Cox regression analyses for OS, high CAR and high SII were associated with shorter OS, with HRs of 2.70 (95% CI, 1.54–4.74; *p* < 0.001) and 2.80 (95% CI, 1.59–4.94; *p* < 0.001), respectively (**Table 1**). In the main multivariable model, high CAR and high SII remained independently associated with shorter OS, with adjusted HRs of 2.36 (95% CI, 1.30–4.27; *p* = 0.005) and 2.58 (95% CI, 1.42–4.69; *p* = 0.002), respectively (**Table 2**).

**Table 2:** Univariable and multivariable Cox regression analyses for overall survival.

Variable	Univariable HR (95% CI)	*p* value	Multivariable HR (95% CI)	*p* value
IMDC intermediate vs favorable	3.24 (0.44–24.03)	0.251	1.81 (0.23–14.04)	0.570
IMDC poor vs favorable	6.90 (0.93–51.28)	0.059	2.33 (0.29–18.86)	0.427
Nephrectomy yes	0.38 (0.22–0.68)	0.001	0.38 (0.21–0.68)	< 0.001
High CAR	2.70 (1.54–4.74)	< 0.001	2.36 (1.30–4.27)	0.005
High SII	2.80 (1.59–4.94)	< 0.001	2.58 (1.42–4.69)	0.002

The multivariable model included IMDC risk classification, nephrectomy status, high CAR, and high SII. High and low CAR were defined as CAR ≥ 0.0731 and CAR < 0.0731, respectively. High and low SII were defined as SII ≥ 533.81 and SII < 533.81, respectively. HR: hazard ratio, CI: confidence interval, IMDC: International Metastatic Renal Cell Carcinoma Database Consortium, CAR:C-reactive protein-to-albumin ratio, SII: systemic immune-inflammation index.

To assess the potential influence of treatment heterogeneity, we performed a sensitivity analysis additionally adjusting for ICI-based first-line therapy. In this model, high CAR and high SII remained independently associated with shorter OS, with adjusted HRs of 2.36 (95% CI, 1.31–4.27; p = 0.004) and 2.50 (95% CI, 1.33–4.70; *p* = 0.004), respectively. Treatment with ICI-based first-line therapy was not significantly associated with OS (adjusted HR, 0.86; 95% CI, 0.32–2.33; *p* = 0.767) (S1 Table).

To address histological heterogeneity, we performed a sensitivity analysis restricted to patients with confirmed clear-cell RCC. In this analysis, high SII remained significantly associated with shorter OS (adjusted HR, 3.39; 95% CI, 1.44–8.00; *p* = 0.005). High CAR showed an association in the same direction but did not reach statistical significance (adjusted HR, 1.95; 95% CI, 0.91–4.21; *p* = 0.087) (S2 Table).

To reduce reliance on dichotomized cutoff values, CAR and SII were analyzed as log-transformed standardized continuous variables. In this sensitivity analysis, both markers remained significantly associated with OS. The adjusted HRs per 1 standard deviation increase were 1.58 for CAR (95% CI, 1.09–2.28; *p* = 0.015) and 1.97 for SII (95% CI, 1.30–2.98; *p* = 0.001) (S3 Table).

### Incremental prognostic performance beyond IMDC

We assessed incremental prognostic performance beyond IMDC using bootstrap internal validation with 300 resamples. The optimism-corrected C-index was 0.641 for the IMDC model and 0.733 for the IMDC+CAR + SII model, corresponding to a modest improvement of 0.092 (Fig 4 and **Table 3**). Because the expanded model included additional variables and the absolute discrimination remained limited, this improvement should be interpreted as exploratory. The time-dependent AUCs for the IMDC+CAR + SII model were 0.728 (95% CI, 0.581–0.875), 0.756 (95% CI, 0.648–0.864), and 0.767 (95% CI, 0.630–0.904) at 1, 3, and 5 years, respectively. The corresponding AUCs for the IMDC model were 0.602 (95% CI, 0.479–0.725), 0.672 (95% CI, 0.571–0.773), and 0.606 (95% CI, 0.476–0.735), respectively (Fig 5(A) and S4 Table). We assessed performance at 1, 3, and 5 years and primarily emphasized the 1- and 3-year results because later time points can be less stable as the number of patients at risk decreases. Prediction error assessed by Brier scores was lower for the IMDC+CAR + SII model than for IMDC alone at 1, 3, and 5 years, with values of 0.120 versus 0.137 at 1 year, 0.204 versus 0.215 at 3 years, and 0.192 versus 0.227 at 5 years (Fig 5(B) and S4 Table). However, because the AUC values remained moderate and the confidence intervals were relatively wide, these findings should be interpreted as exploratory.

**Table 3:** Discrimination for overall survival comparing IMDC with the IMDC plus CAR and SII model.

	IMDC	IMDC+CAR + SII	ΔC-index
C-index	0.641	0.733	0.092

C-index values are optimism-corrected estimates obtained by bootstrap internal validation with 300 resamples. The ΔC-index value represents the optimism-corrected C-index for the IMDC plus CAR and SII model minus that for IMDC alone, with a positive value indicating improved discrimination. IMDC: International Metastatic Renal Cell Carcinoma Database Consortium, CAR: C-reactive protein-to-albumin ratio, SII: systemic immune-inflammation index.

**Fig 4:**
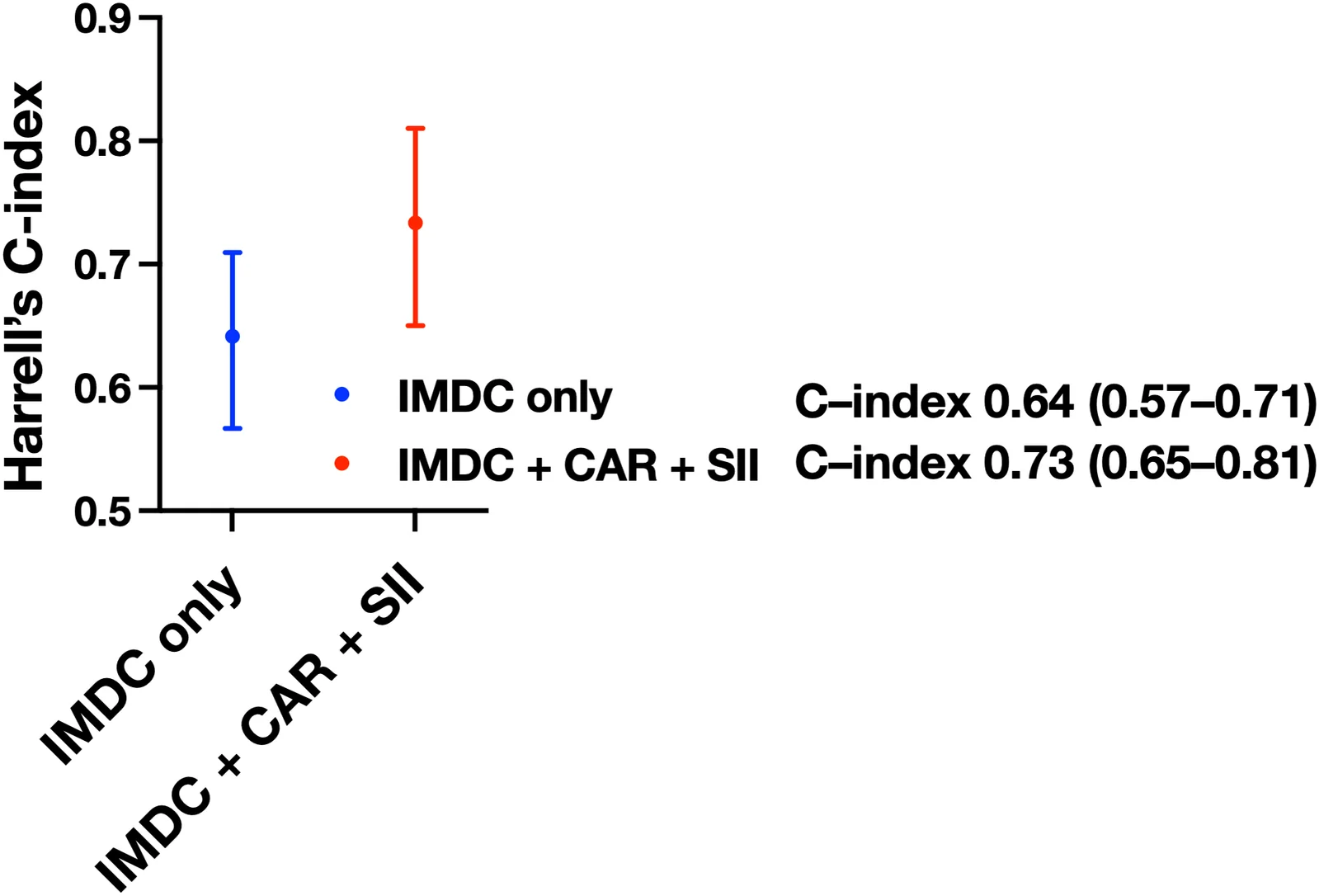
Model discrimination comparing IMDC vs IMDC+CAR + SII for OS. Harrell’s C-index is shown as apparent and bootstrap optimism-corrected estimates. IMDC: International Metastatic Renal Cell Carcinoma Database Consortium, CAR: C-reactive protein-to-albumin ratio, SII: systemic immune-inflammation index, OS: Overall Survival.

**Fig 5:**
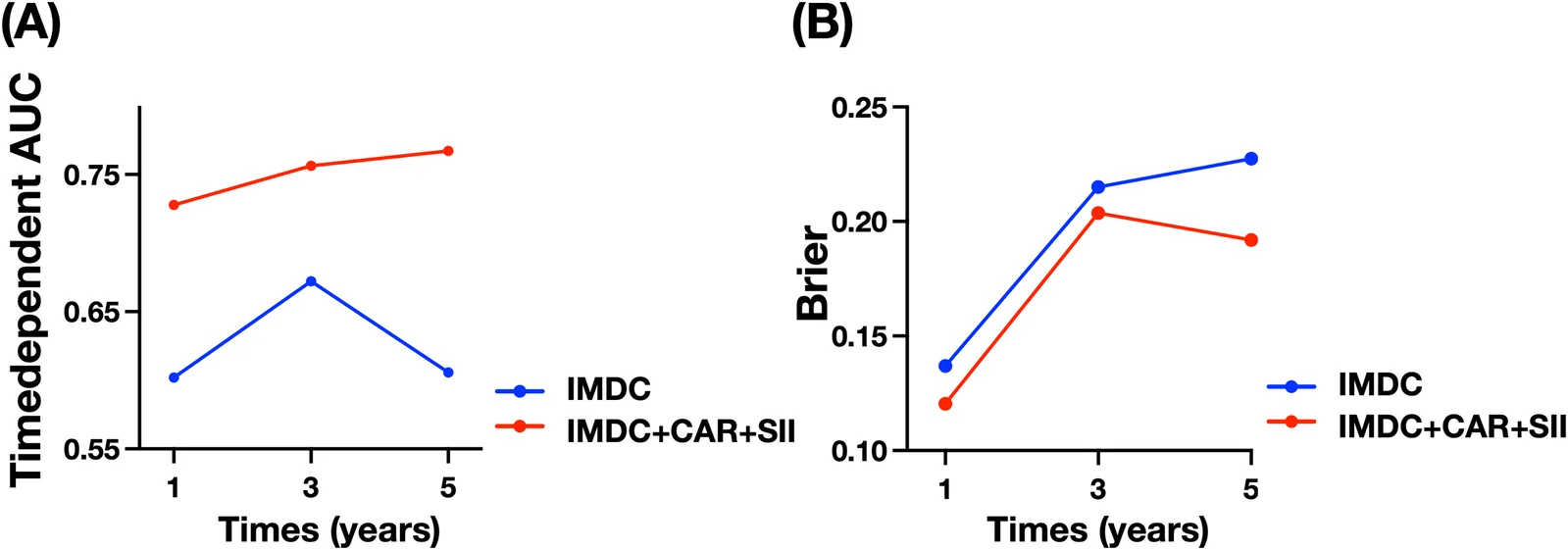
Time-dependent AUC and Brier score for overall survival comparing IMDC with the IMDC plus CAR and SII model. (A) Time-dependent AUC at 1, 3, and 5 years for OS comparing the IMDC model with the IMDC plus CAR and SII model. (B) Brier score at 1, 3, and 5 years for overall survival for the same models, with lower values indicating better overall predictive performance. CAR: C-reactive protein-to-albumin ratio, SII: systemic immune-inflammation index, AUC: area under the curve.

Calibration was assessed for the IMDC model and the IMDC+CAR + SII model at 1, 3, and 5 years. Calibration plots showed generally acceptable agreement between predicted and observed survival probabilities for both models, although some deviation was observed, particularly at later time points (S3 Fig). The calibration slopes were 1.000 (95% CI, 0.363–1.637) for the IMDC model and 1.000 (95% CI, 0.632–1.368) for the IMDC+CAR + SII model (S5 Table). **Decision curve analysis** showed higher net benefit for the IMDC+CAR + SII model than for IMDC alone across clinically relevant threshold probabilities at 1 and 3 years (S2 Fig). Overall, these model performance analyses suggested modest and exploratory improvement after adding CAR and SII to IMDC.

## Discussion

The main finding of this study is that pretreatment CAR and SII are readily available inflammatory biomarkers that may help refine OS risk assessment in patients with mRCC receiving first-line systemic therapy. In particular, patients with elevation of both CAR and SII had the poorest survival, and the addition of these markers to established clinical factors modestly improved prognostic performance. These findings suggest that CAR and SII may provide complementary prognostic information, although their clinical utility requires external validation before implementation in routine practice.

The relationship between cancer and inflammation has been repeatedly documented in both basic and clinical research. Depending on tumor type and context, inflammation may precede malignant transformation or may be induced by tumorigenic changes and subsequently promote tumor progression [12]. Inflammatory signaling in the tumor microenvironment can enhance proliferation, angiogenesis, and metastatic potential, while also impairing antitumor immunity and altering responses to systemic therapies [12]. In RCC, inflammation-related biomarkers have been widely investigated as prognostic indicators, reflecting the biological relevance of host inflammatory status.

Among individual components, both CRP and albumin have been associated with prognosis in RCC. Meta-analyses have reported that elevated CRP is associated with poorer survival and may have clinical utility for risk assessment [24,25]. Similarly, low pretreatment albumin has been reported as an adverse prognostic factor in patients with mRCC treated with tyrosine kinase inhibitors [25,26]. Composite indices that integrate inflammation and nutrition have also been explored, including models that incorporate albumin, CRP, and NLR in RCC [14]. As a simple metric, CAR combines CRP and albumin, and prior work has identified high CAR as an adverse prognostic factor following nephrectomy, with a reported optimal cutoff around 0.08 [27]. The cutoff derived in our study, 0.0731, is close to that value and further supports the relevance of CAR as a prognostic marker in mRCC.

As a composite marker, SII reflects neutrophil- and platelet-driven inflammation relative to lymphocyte-mediated immune competence [17]. Several studies have reported associations between elevated SII and poorer survival in RCC, including metastatic settings [17,18,28,29]. However, there is no consensus on the optimal cutoff value, likely due to differences in patient populations, treatment eras, laboratory conventions, and analytical approaches. In this context, we selected an exploratory cutoff of 533.81 using time-dependent ROC analysis at the prespecified time horizon. This value is broadly consistent with cutoffs reported in prior studies, such as 628 [18] and 730 [17,29]. Using this exploratory threshold, higher SII was associated with shorter OS in our cohort, in line with prior reports.

A key contribution of our study is the evaluation of CAR and SII not only as individual biomarkers but also as a combined stratification approach. While many prior studies have focused on single inflammatory indices, data on combining multiple inflammation-based indices and quantifying their incremental value beyond established risk models such as IMDC remain limited. In our cohort, patients with concurrent elevation of both CAR and SII showed the shortest OS, and the three-group stratification suggested a stepwise worsening of prognosis from neither elevated to one elevated to both elevated. This pattern supports the possibility that CAR and SII capture complementary aspects of host–tumor biology and may offer added prognostic resolution when used together.

From a clinical perspective, CAR and SII may be useful as adjunctive markers to complement, rather than replace, established clinical risk models. Because these indices are inexpensive and readily available from routine pretreatment laboratory data, they may help refine baseline risk assessment, support prognostic discussion, identify patients who may require closer follow-up, and serve as stratification or adjustment factors in future clinical trials. However, the present findings should not be used alone to select systemic therapy or alter treatment decisions.

Because this study was retrospective and observational, CAR and SII should be interpreted as host inflammatory markers rather than mechanistic evidence identifying a specific cause of inflammation. Peripheral inflammatory markers cannot distinguish whether inflammation is driven by tumor biology, infection, comorbid inflammatory conditions, nutritional deterioration, sarcopenia, or treatment-related changes [30,31]. Therefore, the present findings do not directly support a specific anti-inflammatory or preventive intervention. Future prospective studies should evaluate whether longitudinal changes in CAR and SII reflect treatment response, tumor progression, infection, comorbidities, nutritional deterioration, sarcopenia, or other modifiable inflammatory states [32].

This study has several strengths. It evaluated multiple inflammatory and nutritional indices in a well-characterized cohort of patients with metastatic renal cell carcinoma treated in routine practice, and all variables included in the analyses were complete, enabling transparent modelling without imputation. We prespecified OS as the primary outcome and PFS as the secondary outcome, and we assessed discrimination using time-dependent receiver operating characteristic analyses and clinical utility using decision curve analysis.

Nevertheless, several limitations should be considered. First, this was a single-center retrospective study conducted in Japan, and the cohort included patients treated across different therapeutic eras. Because only 24 patients received ICI-based first-line therapy, the findings may not be directly generalizable to contemporary cohorts treated predominantly with current ICI-based combination regimens. In addition, treatment regimens, subsequent-line therapies, comorbidity burden, and unmeasured tumor-related factors may have influenced survival. Second, residual confounding related to baseline clinical factors cannot be excluded. Although nephrectomy status was included as a covariate in the main multivariable model, the absence of nephrectomy may reflect patient selection and disease severity. In the present cohort, nephrectomy was not performed in 36 patients for non-uniform clinical reasons, including advanced metastatic burden, limited suitability for surgery, treatment strategies prioritizing systemic therapy, or lack of indication for cytoreductive nephrectomy at the treating physician’s discretion. The IMDC risk classification was not statistically significant in the main multivariable model, which may reflect the small favorable-risk group, potential overlap between IMDC components and systemic inflammatory markers, and limited model stability. Performance status and histology were not added separately to the primary model because performance status is incorporated in the IMDC risk classification, only two patients had KPS < 80, and histological subtype was unknown in 23 patients. Although the clear-cell RCC-only sensitivity analysis supported the association between high SII and shorter OS, histological heterogeneity should be evaluated further in larger cohorts with complete histological information. Third, the prognostic model findings should be interpreted cautiously. The observed improvement in prognostic performance after adding CAR and SII to IMDC was modest, the expanded model included additional variables, and the absolute predictive performance remained limited. Calibration assessment suggested generally acceptable agreement between predicted and observed survival probabilities, but these findings are limited by the modest sample size and the small number of patients at risk at later time points. In addition, the CAR and SII cutoffs were exploratory and derived from a prespecified five-year horizon. Although the CAR cutoff of 0.0731 was close to a previously reported cutoff of 0.08 in metastatic RCC [27], and the SII cutoff of 533.81 was broadly comparable with a previously reported SII cutoff of 730 [29], these thresholds require external validation before application to other cohorts. The continuous-variable sensitivity analysis supported that the prognostic associations of CAR and SII were not solely dependent on the selected cutoff values.

Future multicenter studies with prospective data collection and external validation are needed to confirm these findings and refine clinically implementable risk models.

In conclusion, our study suggests that CAR and SII are readily obtainable indices associated with prognosis in mRCC. Their combined use may modestly improve risk stratification beyond established clinical models. Further multicenter validation is warranted to confirm these findings and clarify how CAR and SII can be best implemented in clinical decision-making.

## Supporting information

S1 Table Sensitivity analysis additionally adjusted for ICI-based first-line therapy. The model included IMDC risk classification, nephrectomy status, high CAR, high SII, and ICI-based first-line therapy. High CAR was defined as CAR ≥ 0.0731, and high SII was defined as SII ≥ 533.81. HR: hazard ratio, CI: confidence interval, IMDC: International Metastatic Renal Cell Carcinoma Database Consortium, CAR:C-reactive protein/albumin ratio, SII: systemic immune-inflammation index, ICI: Immune checkpoint inhibitor.
(PDF)

S2 Table The clear-cell RCC-only sensitivity analysis was restricted to patients with confirmed clear-cell RCC. The model included IMDC risk classification, nephrectomy status, high CAR, and high SII. High CAR was defined as CAR ≥ 0.0731, and high SII was defined as SII ≥ 533.81. HR: hazard ratio, CI: confidence interval, IMDC: International Metastatic Renal Cell Carcinoma Database Consortium, CAR:C-reactive protein/albumin ratio, SII: systemic immune-inflammation index.
(PDF)

S3 Table Sensitivity analysis treating CAR and SII as log-transformed standardized continuous variables for OS. The multivariable Cox proportional hazards model included IMDC risk classification, nephrectomy status, log-transformed CAR, and log-transformed SII. CAR and SII were log-transformed and standardized, and hazard ratios are shown per 1 standard deviation increase. HR: hazard ratio, CI: confidence interval, IMDC: International Metastatic Renal Cell Carcinoma Database Consortium, CAR:C-reactive protein/albumin ratio, SII: systemic immune-inflammation index.
(PDF)

S4 Table Time-dependent AUC and Brier score for overall survival comparing IMDC with the IMDC plus CAR and SII model. Time-dependent AUC and Brier scores were calculated for overall survival at prespecified time points of 1, 3, and 5 years. Higher AUC indicates better discrimination, whereas lower Brier score indicates better overall predictive performance.CI: confidence interval, IMDC: International Metastatic Renal Cell Carcinoma Database Consortium, CAR: C-reactiveprotein-to-albumin ratio, SII: systemic immune-inflammation index.
(PDF)

S5 Table Calibration slopes for the IMDC model and the IMDC+CAR + SII model. Calibration slopes were calculated using the Cox linear predictor. Values are shown with 95% confidence intervals. IMDC+CAR + SII indicates the model incorporating IMDC risk classification, CAR, and SII. CI: confidence interval, IMDC: International Metastatic Renal Cell Carcinoma Database Consortium, CAR:C-reactive protein/albumin ratio, SII: systemic immune-inflammation index.
(PDF)

S1 Fig Time-dependent ROC curves for NLR, PLR, and MLR for OS at 1, 3, and 5 years. Time-dependent receiver operating characteristic curves for overall survival at 1, 3, and 5 years are shown for inflammatory indices. The curves compare the discriminative ability of each index at the corresponding time point, and the area under the curve is provided in the figure. High and low CAR were defined as CAR ≥ 0.0731 and CAR < 0.0731, respectively. High and low SII were defined as SII ≥ 533.81 and SII < 533.81, respectively. NLR: neutrophil-to-lymphocyte ratio, PLR: platelet-to-lymphocyte ratio, MLR: monocyte-to-lymphocyte ratio index, AUC: area under the curve.
(PDF)

S2 Fig Decision curve analysis showing net benefit across threshold probabilities at 1 and 3 years for overall survival. The net benefit of the IMDC model is compared with that of the IMDC plus CAR and SII model, along with default strategies of treating all patients and treating no patients. Higher net benefit indicates greater clinical utility across the corresponding threshold range. DCA: decision curve analysis, IMDC: International Metastatic Renal Cell Carcinoma Database Consortium, CAR: C-reactive protein-to-albumin ratio; SII: systemic immune-inflammation index.
(PNG)

S3 Fig Calibration plots for the IMDC and IMDC+CAR + SII models. Calibration plots for the IMDC model and the IMDC+CAR + SII model at 1, 3, and 5 years are shown. The dashed diagonal line represents perfect calibration. IMDC+CAR + SII indicates the model incorporating IMDC risk classification, CAR, and SII. IMDC: International Metastatic Renal Cell Carcinoma Database Consortium, CAR: C-reactive protein-to-albumin ratio; SII: systemic immune-inflammation index.
(PNG)
