# Supplementary material for: Clinical utility of combining the C-reactive protein-to-albumin ratio and systemic immune-inflammation index for prognostic assessment in metastatic renal cell carcinoma
Source: PLoS One. 2026 Oct 7;21(10):e0345724. doi: 10.1371/journal.pone.0345724 (PMC13645026; doi:10.1371/journal.pone.0345724)
Supplement: Supplementary file 1 [file pone.0345724.s001.pdf]

Parameter	HR (95% CI)	*p* value
**IMDC risk classification, intermediate vs favorable**	1.85 (0.24–14.36)	0.558
**IMDC risk classification, poor vs favorable**	2.38 (0.29–19.32)	0.417
**Nephrectomy, yes**	0.37 (0.20–0.68)	0.001
**CAR, ≥0.0731**	2.36 (1.31–4.27)	0.004
**SII, ≥533.81**	2.50 (1.33–4.70)	0.004
**ICI-based first-line therapy, yes**	0.86 (0.32–2.33)	0.767
