# Supplementary material for: Clinical utility of combining the C-reactive protein-to-albumin ratio and systemic immune-inflammation index for prognostic assessment in metastatic renal cell carcinoma
Source: PLoS One. 2026 Oct 7;21(10):e0345724. doi: 10.1371/journal.pone.0345724 (PMC13645026; doi:10.1371/journal.pone.0345724)
Supplement: Supplementary file 2 [file pone.0345724.s002.pdf]

Variable	Adjusted HR (95% CI)	*p* val
**IMDC intermediate vs favorable**	1.56 (0.19–12.74)	0.676
**IMDC poor vs favorable**	1.77 (0.20–15.65)	0.605
**Nephrectomy yes**	0.30 (0.09–0.94)	0.038
**High CAR**	1.95 (0.91–4.21)	0.087
**High SII**	3.39 (1.44–8.00)	0.005
