# Supplementary material for: Clinical utility of combining the C-reactive protein-to-albumin ratio and systemic immune-inflammation index for prognostic assessment in metastatic renal cell carcinoma
Source: PLoS One. 2026 Oct 7;21(10):e0345724. doi: 10.1371/journal.pone.0345724 (PMC13645026; doi:10.1371/journal.pone.0345724)
Supplement: Supplementary file 3 [file pone.0345724.s003.pdf]

Variable	Adjusted HR (95% CI)	*p* value
**IMDC intermediate vs favorable**	1.15 (0.13-9.88)	0.900
**IMDC poor vs favorable**	1.07 (0.12-9.75)	0.953
**Nephrectomy, yes**	0.40 (0.22-0.72)	0.003
**Log-transformed CAR, per 1 SD increase**	1.58 (1.09-2.28)	0.015
**Log-transformed SII, per 1 SD increase**	1.97 (1.30-2.98)	0.001
