# Supplementary material for: Clinical utility of combining the C-reactive protein-to-albumin ratio and systemic immune-inflammation index for prognostic assessment in metastatic renal cell carcinoma
Source: PLoS One. 2026 Oct 7;21(10):e0345724. doi: 10.1371/journal.pone.0345724 (PMC13645026; doi:10.1371/journal.pone.0345724)
Supplement: Supplementary file 4 [file pone.0345724.s004.pdf]

		IMDC (95% CI)	IMDC+CAR+SII (95% CI)
AUC	1 year	0.602 (0.479–0.725)	0.728 (0.581–0.875)
	3 years	0.672 (0.571–0.773)	0.756 (0.648–0.864)
	5 years	0.606 (0.476–0.735)	0.767 (0.630–0.904)
Brier	1 year	0.137 (0.092–0.182)	0.120 (0.080–0.161)
	3 years	0.215 (0.185–0.245)	0.204 (0.161–0.246)
	5 years	0.227 (0.185–0.270)	0.192 (0.147–0.237)
