# Supplementary material for: Clinical utility of combining the C-reactive protein-to-albumin ratio and systemic immune-inflammation index for prognostic assessment in metastatic renal cell carcinoma
Source: PLoS One. 2026 Oct 7;21(10):e0345724. doi: 10.1371/journal.pone.0345724 (PMC13645026; doi:10.1371/journal.pone.0345724)
Supplement: Supplementary file 5 [file pone.0345724.s005.pdf]

Model	Calibration slope (95% CI)
**IMDC model**	1.000 (0.363–1.637)
**IMDC+CAR+SII model**	1.000 (0.632–1.368)
