# Supplementary material for: Clinical utility of combining the C-reactive protein-to-albumin ratio and systemic immune-inflammation index for prognostic assessment in metastatic renal cell carcinoma
Source: PLoS One. 2026 Oct 7;21(10):e0345724. doi: 10.1371/journal.pone.0345724 (PMC13645026; doi:10.1371/journal.pone.0345724)
Supplement: Supplementary file 6 [file pone.0345724.s006.pdf]

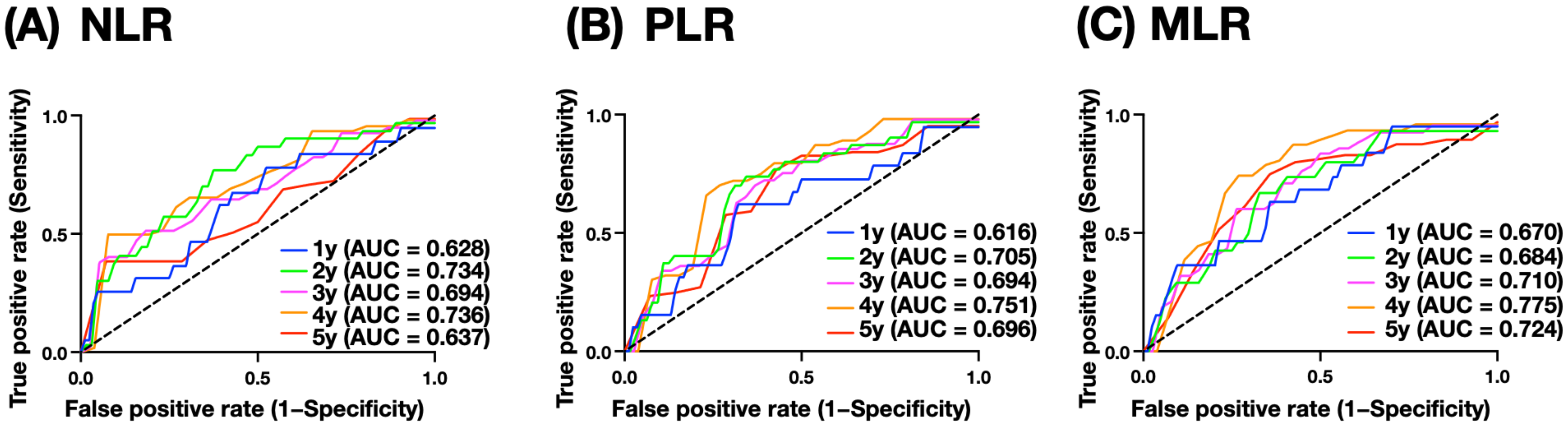
(A) NLR
True positive rate (Sensitivity)
False positive rate (1−Specificity)
1y (AUC = 0.628)
2y (AUC = 0.734)
3y (AUC = 0.694)
4y (AUC = 0.736)
5y (AUC = 0.637)
(B) PLR
True positive rate (Sensitivity)
False positive rate (1−Specificity)
1y (AUC = 0.616)
2y (AUC = 0.705)
3y (AUC = 0.694)
4y (AUC = 0.751)
5y (AUC = 0.696)
(C) MLR
True positive rate (Sensitivity)
False positive rate (1−Specificity)
1y (AUC = 0.670)
2y (AUC = 0.684)
3y (AUC = 0.710)
4y (AUC = 0.775)
5y (AUC = 0.724)
