# Supplementary material for: Clinical utility of combining the C-reactive protein-to-albumin ratio and systemic immune-inflammation index for prognostic assessment in metastatic renal cell carcinoma
Source: PLoS One. 2026 Oct 7;21(10):e0345724. doi: 10.1371/journal.pone.0345724 (PMC13645026; doi:10.1371/journal.pone.0345724)
Supplement: Peer review history

# Peer review: Decision Letter 0

**DOI:** 10.1371/journal.pone.0345724.r001

**Authors:** Sorana Bolboacă

26 Jun 2026

Dear Dr. Nagata,

Thank you for submitting your manuscript to PLOS ONE. After careful consideration, we feel that it has merit but does not fully meet PLOS ONE’s publication criteria as it currently stands. Therefore, we invite you to submit a revised version of the manuscript that addresses the points raised during the review process.

==============================

**ACADEMIC EDITOR:**

- Provide a structured abstract. Do not include in the abstract information regarding the statistical methods used. Provide also the associated 95%CI.
- It is unclear why you provided incidence on the US population but your study is on diferent population.
- "Hematuria, flank pain, and abdominal masses are the classical three signs" this is unspecific.
- Do not start a sentence with an abbreviation.
- What "A certain number of" mean?
- Why is the presentation of treatment options useful for your manuscript? Which population are these treatment options available? (US population or your target population?).
- "have been identified as candidate prognostic biomarkers" this is too generic; please be more specific. Comment applies to any list markers.
- "but the optimal cutoff values remain unclear" this is not true.
- "inflammatory and nutritional markers" which one? Be specific.
- The state of the art missed the gaps in the scientific literature related to the targeted biomarkers.
- Provide the approval date and number for the ethics committee.
- "PNI, GNRI, and mGPS were calculated as previously reported." Reference is missing; please also provide the calculation formula.
- "defined as 40 and 98," these cutoff were obtained on the target population?
- "Patient and Public Involvement" this section should be deleted.
- "OS was defined as the time from initiation of first-line systemic therapy" why not from the diagnosis?
- "AI-assisted technologies" should be moved to the end of the manuscript in the section of declarations.
- A result must stay either in text, in a table, or in a figure. Do not duplicate the results.
- Table 1 should also include univariable analysis for targeted outcomes.
- Transparency in multivariable analysis is missing. It is unclear how the multivariable analysis was conducted.
- Cox regression without including covariates is misleading. This is a major issue.
- The increase in C-index (Table 3) is given by considering more variable. Which is the clinical relevance, considering that the values are low?
- AUC should be reported with 95% confidence intervals. As are looking, none AUC show clinical relevance.
- Discussion - Do not make a summary of your study. Start this section by presenting the main conclusion of your study.
- The clinical relevance of the reported results is not appropriately discussed.
- The generalizability of the reported results is not appropriately discussed.

==============================

Please submit your revised manuscript by Aug 10 2026 11:59PM. If you will need more time than this to complete your revisions, please reply to this message or contact the journal office at plosone@plos.org. When you're ready to submit your revision, log on to https://www.editorialmanager.com/pone/ and select the 'Submissions Needing Revision' folder to locate your manuscript file.

Please include the following items when submitting your revised manuscript:

- A letter that responds to each point raised by the academic editor and reviewer(s). You should upload this letter as a separate file labeled 'Response to Reviewers'.
- A marked-up copy of your manuscript that highlights changes made to the original version. You should upload this as a separate file labeled 'Revised Manuscript with Track Changes'.
- An unmarked version of your revised paper without tracked changes. You should upload this as a separate file labeled 'Manuscript'.

If you would like to make changes to your financial disclosure, please include your updated statement in your cover letter. Guidelines for resubmitting your figure files are available below the reviewer comments at the end of this letter.

If applicable, we recommend that you deposit your laboratory protocols in protocols.io to enhance the reproducibility of your results. Protocols.io assigns your protocol its own identifier (DOI) so that it can be cited independently in the future. For instructions see: https://journals.plos.org/plosone/s/submission-guidelines#loc-laboratory-protocols. Additionally, PLOS ONE offers an option for publishing peer-reviewed Lab Protocol articles, which describe protocols hosted on protocols.io. Read more information on sharing protocols at https://plos.org/protocols?utm_medium=editorial-email&utm_source=authorletters&utm_campaign=protocols.

As the corresponding author, your ORCID iD is verified in the submission system and will appear in the published article. PLOS supports the use of ORCID, and we encourage all coauthors to register for an ORCID iD and use it as well. Please encourage your coauthors to verify their ORCID iD within the submission system before final acceptance, as unverified ORCID iDs will not appear in the published article. *Only*  the individual author can complete the verification step; PLOS staff *cannot*  verify ORCID iDs on behalf of authors.

We look forward to receiving your revised manuscript.

Kind regards,

Sorana D. Bolboacă, Ph.D., M.Sc., M.D.

Academic Editor

PLOS One

**Journal Requirements:**

When submitting your revision, we need you to address these additional requirements.

1. Please ensure that your manuscript meets PLOS ONE's style requirements, including those for file naming. The PLOS ONE style templates can be found at

https://journals.plos.org/plosone/s/file?id=wjVg/PLOSOne_formatting_sample_main_body.pdf and

https://journals.plos.org/plosone/s/file?id=ba62/PLOSOne_formatting_sample_title_authors_affiliations.pdf

2. We kindly ask you to address the following concerns:

We note that you have declared the use of generative AI in the preparation of your manuscript. Please note that contributions by artificial intelligence (AI) tools and technologies to a study or to an article’s contents must be clearly reported in a dedicated section of the Methods, or in the Acknowledgements section for article types lacking a Methods section. This section should include the name(s) and version(s) of any tools used, a description of how the authors used the tool(s) and evaluated the validity of the tool’s outputs, and a clear statement of which aspects of the study, article contents, data, or supporting files were affected/generated by AI tool usage (https://journals.plos.org/plosone/s/ethical-publishing-practice#loc-artificial-intelligence-tools-and-technologies).

Please therefore revise your manuscript to provide all necessary information to meet these requirements.

3. In the online submission form, you indicated that your data is available only on request from a third party. Please note that your Data Availability Statement is currently missing the contact details for the third party, such as an email address or a link to where data requests can be made. Please update your statement with the missing information.

4. If the reviewer comments include a recommendation to cite specific previously published works, please review and evaluate these publications to determine whether they are relevant and should be cited. There is no requirement to cite these works unless the editor has indicated otherwise.

[Note: HTML markup is below. Please do not edit.]

Reviewers' comments:

Reviewer's Responses to Questions

**Comments to the Author**

1. Is the manuscript technically sound, and do the data support the conclusions?

Reviewer #1: Yes

Reviewer #2: Yes

Reviewer #3: Yes

Reviewer #4: Yes

**********

2. Has the statistical analysis been performed appropriately and rigorously?

Reviewer #1: No

Reviewer #2: I Don't Know

Reviewer #3: Yes

Reviewer #4: Yes

**********

3. Have the authors made all data underlying the findings in their manuscript fully available?

The PLOS Data policy

Reviewer #1: No

Reviewer #2: Yes

Reviewer #3: Yes

Reviewer #4: Yes

**********

4. Is the manuscript presented in an intelligible fashion and written in standard English?

Reviewer #1: Yes

Reviewer #2: Yes

Reviewer #3: Yes

Reviewer #4: Yes

**********

Reviewer #1: OVERALL ASSESSMENT:

This retrospective single-center study investigates the prognostic value of two inflammation-based biomarkers—C-reactive protein-to-albumin ratio (CAR) and systemic immune-inflammation index (SII)—in patients with metastatic renal cell carcinoma (mRCC). 117 patients treated with first-line systemic therapy between 2001 and 2022 were included. The authors evaluated pretreatment CAR and SII using time-dependent ROC analyses to define optimal cutoffs (CAR: 0.0731; SII: 533.81), followed by Kaplan–Meier survival analyses and Cox proportional hazards models adjusted for IMDC risk classification and nephrectomy status. The authors conclude that CAR and SII are inexpensive, readily available biomarkers that may enhance prognostic stratification beyond established IMDC criteria, though external validation is required.

MAJOR COMMENTS:

1. Clarify treatment heterogeneity and adjust if possible. Provide stratified analyses (e.g., TKI vs ICI era), or alternatively, include treatment type in multivariable models or sensitivity analyses.

2. Justify and contextualize cutoff values. Emphasize that cutoffs are exploratory, compare quantitatively with prior literature, and consider reporting continuous-variable analyses alongside dichotomized ones.

3. Strengthen multivariable modeling by providing a rationale for excluding key variables and considering adding at least performance status (continuous or categorical) and histology (clear vs non-clear). Report the number of events per variable (EPV).

4. Although discrimination (C-index) and Brier score are reported, please provide calibration assessment and include calibration plots or at least calibration slope/CITL.

5. A ΔC-index of 0.092 is notable but requires contextual interpretation of whether it is clinically meaningful or not. Compare to thresholds used in prognostic model evaluation.

6. Expand on clinical applicability: How would CAR/SII change decision-making? Risk-adapted therapy? Surveillance? Trial stratification?

7. Provide a clearer mechanism for data access. Consider anonymized dataset sharing if possible.

MINOR COMMENTS:

1. The language is generally clear, but minor grammatical inconsistencies persist (likely from mixed editing sources). There is some redundancy in the Introduction and Discussion.

2. In Figure 1 (page 27), multiple ROC curves are overlaid. Consider simplifying or separating panels for readability.

3. In Figure 2 (page 28), median survival annotations are helpful but visually dense.

4. Use consistent formatting for CAR and SII (e.g., “high vs low” vs “≥ cutoff”).

5. Please include number at risk tables (already present, but ensure clarity) and exact p-values instead of thresholds where possible.

6. IMDC appears non-significant in multivariable analysis. This is unexpected and should be discussed explicitly.

Reviewer #2: Thank you to the authors for submitting this manuscript describing a study in which clinical outcomes of mRCC over 5yrs in a single centre were studied in the context of two inflammatory indices (CAR & SII). I have minor queries:

1) There are very occasional minor, unusual turns of phrase for correction.

2) Fig 1 - the performance of CAR degrades somewhat over time compared to SII, which is well preserved. Can the authors explore this?

3) Are the authors able to explore in practical terms the impacts on outcomes with either standard deviations (or deciles/quartiles) of the variance of the CAR & SII?

4) Whilst this is further observational supportive evidence for a role of inflammation in RCC cancerogenesis, this remains a somewhat vague & impersonal association only identified in retrospect and without a well-identified cause of inflammation. Are there any future therapeutic or preventative approaches that the authors might suggest exploring?

Reviewer #3: Congratulations to the authors on this interesting study. However, I have two concerns regarding the presented results.

1. In the Results section, the authors report that 81 patients underwent nephrectomy. Could the authors clarify why the remaining patients in the cohort did not undergo nephrectomy? This information was not clearly presented in the manuscript and may be important for understanding patient selection and potential sources of bias.

2. According to the Results table, 15 patients had a non–clear cell histological subtype and 23 patients had an unknown histological subtype. Nevertheless, these patients were included in the analyses. Could the authors clarify how the CAR and SII analyses were performed and interpreted for these patients, given that they did not have confirmed metastatic clear cell renal cell carcinoma (mRCC)?

It is possible that this issue has already been addressed by the authors; however, I was unable to identify a clear description in the manuscript. If such analyses were not performed, I would suggest conducting a sensitivity analysis excluding patients with non–clear cell histology and those with unknown histological subtype, in order to evaluate the robustness of the findings and the potential impact of histological heterogeneity on the reported associations.

Reviewer #4: The term outcomes has been used a lot throughout the manuscript. It has not been clearly defined what outcome exactly is/are?

Especially the starting portion of the discussion- mentiones about clinical outcomes which seems a vague term in the contect of the discussion.

**********

what does this mean?). If published, this will include your full peer review and any attached files.

If you choose “no”, your identity will remain anonymous but your review may still be made public.

**Do you want your identity to be public for this peer review?**  For information about this choice, including consent withdrawal, please see our Privacy Policy

Reviewer #1: **Yes:**  Murat Akand

Reviewer #2: No

Reviewer #3: No

Reviewer #4: **Yes:**  Hari Naga Garapati

**********

[NOTE: If reviewer comments were submitted as an attachment file, they will be attached to this email and accessible via the submission site. Please log into your account, locate the manuscript record, and check for the action link "View Attachments". If this link does not appear, there are no attachment files.]

To ensure your figures meet our technical requirements, please review our figure guidelines: https://journals.plos.org/plosone/s/figures

You may also use PLOS’s free figure tool, NAAS, to help you prepare publication quality figures: https://journals.plos.org/plosone/s/figures#loc-tools-for-figure-preparation.

NAAS will assess whether your figures meet our technical requirements by comparing each figure against our figure specifications.

# Author comment: Author response to Decision Letter 1

**DOI:** 10.1371/journal.pone.0345724.r002

6 Aug 2026

Reply to Academic Editor

We thank the Academic Editor for the careful review and constructive comments. We have revised the manuscript and performed additional analyses in response to these comments, as detailed below.

Comments from Academic Editor:

Provide a structured abstract. Do not include in the abstract information regarding the statistical methods used. Provide also the associated 95%CI.

Response: We thank the Academic Editor for this comment. We have revised the Abstract to a structured format with Background, Methods, Results, and Conclusions subsections. We have also removed detailed statistical descriptions from the Abstract and added 95% confidence intervals for the main effect estimates.

The details of the revision are as follows: detailed statistical methods have been removed from the Abstract, and 95% confidence intervals for the main prognostic estimates have been added.

It is unclear why you provided incidence on the US population but your study is on diferent population.

Response: We agree with your comment. Because the present study was conducted in a Japanese single-center cohort, we have removed the US-specific incidence statement from the Introduction. The opening paragraph has been revised to describe the global burden of kidney cancer using worldwide estimates and to explain the relevance of evaluating prognostic markers in Asian clinical cohorts.

The details of the revision are as follows: The first paragraph of the Introduction has been revised (page 3, lines 52–57).

"Hematuria, flank pain, and abdominal masses are the classical three signs" this is unspecific.

Response: We agree that this statement was not specific and was not essential to the rationale of the present study. Therefore, we have removed this sentence from the Introduction and revised the paragraph to focus more directly on the clinical challenge of prognostic assessment in mRCC.

The details of the revision are as follows: The sentence regarding the classical triad of RCC symptoms has been deleted from the Introduction (page 3, lines 52–59).

Do not start a sentence with an abbreviation.

Response: We thank the Academic Editor for pointing this out. We reviewed the manuscript and revised sentences that began with abbreviations such as RCC, mRCC, CAR, SII, OS, and PFS, where appropriate.

The details of the revision are as follows: The manuscript has been revised to avoid beginning sentences with abbreviations.

What "A certain number of" mean?

Response: We agree that the phrase “A certain number of” was vague. We have removed this phrase and revised the sentence to more clearly describe metastatic presentation.

The relevant sentence in the Introduction has been revised as follows: “However, some patients present with metastatic disease at diagnosis, whereas others develop recurrence or metastasis after treatment for localized disease.” (page 3, lines 60–61).

Why is the presentation of treatment options useful for your manuscript? Which population are these treatment options available? (US population or your target population?).

Response: We agree that the previous description of the treatment options was too broad and not sufficiently linked to the rationale of the study. We have revised this section to describe the evolution of systemic therapy for mRCC in general, rather than treatment options specific to the US population. We also clarified that this background is relevant because the prognosis remains heterogeneous in the metastatic setting despite advances in targeted therapy and immune checkpoint inhibitor-based regimens, supporting the need for accessible prognostic biomarkers at the initiation of first-line systemic therapy.

The details of the revision are as follows: The treatment-related paragraph in the Introduction has been revised to connect the evolution of systemic therapy with the need for prognostic stratification at the initiation of first-line systemic therapy (page 3, lines 60–65).

"have been identified as candidate prognostic biomarkers" this is too generic; please be more specific. Comment applies to any list markers.

Response: We agree with your comment. We revised the Introduction to avoid the generic phrase “candidate prognostic biomarkers” and to more precisely describe the evaluated markers. In particular, we now state that CAR integrates C-reactive protein and albumin, thereby capturing systemic inflammation and nutritional status, whereas SII incorporates neutrophil, platelet, and lymphocyte counts and reflects the balance between inflammation, thrombocytosis, and immune status.

The details of the revision are as follows: The Introduction has been revised to provide more specific descriptions of CAR, SII, and related inflammatory or nutrition-related indices and to clarify why CAR and SII were selected as the main biomarkers of interest (page 4, lines 75–93).

"but the optimal cutoff values remain unclear" this is not true.

Response: We agree with the Academic Editor that the original statement was inaccurate because the cutoff values for CAR, SII, and related inflammatory indices have been previously reported. Therefore, we have revised the manuscript to avoid stating that the optimal cutoff values remain unclear. Instead, we now state that the reported cutoff values vary across studies and that this variability may reflect differences in patient populations, treatment settings, laboratory conventions, time horizons, and statistical methods.

In addition, we clarified that the cutoff values used in the present study were exploratory and cohort-derived. Specifically, exploratory cutoffs for CAR, NLR, PLR, MLR, and SII were derived using time-dependent ROC analysis for OS at the pre-specified 5-year time horizon and the Youden index. In contrast, PNI and GNRI were dichotomized using previously reported cutoff values and were not derived from the present cohort.

The details of the revision are as follows: The Introduction has been revised to state that the reported cutoff values vary across studies, and the Material and methods section has been revised to clarify that the cutoffs for CAR, NLR, PLR, MLR, and SII were exploratory and cohort-derived, whereas the cutoffs for PNI and GNRI were based on previous reports (page 4, lines 93–95; page 6, lines 133–138).

"inflammatory and nutritional markers" which one? Be specific.

Response: We agree with your comment. We have revised the Introduction to explicitly name the inflammatory and nutrition-related indices discussed in this study, including CAR, NLR, PLR, MLR, SII, mGPS, PNI, and GNRI. We also clarified that CAR and SII were the main biomarkers of interest and described what each marker reflects.

The details of the revision are as follows: The Introduction now lists the inflammatory and nutrition-related indices and explains that CAR integrates C-reactive protein and albumin, thereby capturing systemic inflammation and nutritional status, whereas SII incorporates neutrophil, platelet, and lymphocyte counts and reflects the balance between inflammation, thrombocytosis, and immune status (page 4, lines 77–92).

The state of the art missed the gaps in the scientific literature related to the targeted biomarkers.

Response: We thank the Academic Editor for this important comment. We have revised the Introduction to clarify the specific gap addressed in this study. Although CAR and SII have been investigated as prognostic biomarkers in RCC, fewer studies have evaluated their combined prognostic value and incremental contribution beyond established risk classification systems such as IMDC, particularly in patients receiving first-line systemic therapy for mRCC.

The details of the revision are as follows: The final part of the Introduction has been revised to clarify the scientific gap and study objective. Specifically, we now state that the incremental prognostic value of combining CAR and SII with established clinical factors has not been fully clarified and that limited data are available regarding whether their combined use provides additional prognostic information supported by model performance measures, including discrimination, prediction error, calibration, and decision curve analysis (page 4, lines 92–99).

Provide the approval date and number for the ethics committee.

Response: We thank the Academic Editor for this comment. We have added the ethics approval date alongside the approval number in the Material and methods section.

The details of the revision are as follows: The ethics statement in the Material and methods section has been revised to include both the approval date and approval number: This study was approved by the Ethics Committee of Juntendo University on September 28, 2023 (E23-0275-H01). (page 5, lines 108–109).

"PNI, GNRI, and mGPS were calculated as previously reported." Reference is missing; please also provide the calculation formula.

Response: We thank the Academic Editor for this helpful comment. We have revised the Methods section to include the calculation formulas for PNI, GNRI, and mGPS and added the corresponding references.

The PNI was calculated as 10 × serum albumin concentration (g/dL) + 0.005 × total lymphocyte count (/mm³). The GNRI was calculated as 14.89 × serum albumin concentration (g/dL) + 41.7 × body weight/ideal body weight. Ideal body weight was calculated as 22 × height² (m²), and the body weight/ideal body weight ratio was set to 1 when body weight exceeded ideal body weight. The mGPS was assigned according to CRP and serum albumin levels.

The details of the revision are as follows: The Methods section has been revised to include the formulas and references for PNI, GNRI, and mGPS (page 5, lines 119–126).

"defined as 40 and 98," these cutoff were obtained on the target population?

Response: We thank the Academic Editor for this comment. We clarified that the cutoffs for PNI and GNRI were not derived from the present cohort but based on previously reported values. Specifically, PNI and GNRI were dichotomized using cutoff values of 40 and 98, respectively.

The details of the revision are as follows: The “Determination of cutoff values for inflammatory and nutritional markers” section has been revised to clarify that PNI and GNRI were dichotomized using previously reported cutoff values and not derived from the present cohort (page 6, lines 135–137).

"Patient and Public Involvement" this section should be deleted.

Response: We agree with the comment and have deleted the Patient and Public Involvement section from the revised manuscript.

The details of the revision are as follows: The Patient and Public Involvement section has been removed.

"OS was defined as the time from initiation of first-line systemic therapy" why not from the diagnosis?

Response: We thank the Academic Editor for this comment. We defined OS as the time from the initiation of first-line systemic therapy because CAR and SII were measured immediately before systemic therapy, and this study aimed to evaluate their prognostic value at the time of treatment decision-making. This approach aligns the biomarker assessment time point with the start of survival follow-up and reduces the heterogeneity related to the interval between diagnosis and initiation of systemic therapy.

To address the Editor’s concern, we additionally performed a sensitivity analysis using the OS calculated from the date of diagnosis. In this analysis, high CAR and SII remained significantly associated with shorter OS, and the results were consistent with those of the main analysis. Because this analysis was performed to confirm the robustness of the time-origin definition and did not change the interpretation of the study, we have presented the results in this response rather than adding an additional supplementary table to the manuscript.

The details of the revision are as follows: The Material and methods section has been revised to clarify the rationale for defining OS from initiation of first-line systemic therapy (page 6, lines 143–146). Sensitivity analysis using the OS calculated from the date of diagnosis is presented below.

Variable Adjusted HR (95% CI) p-value

IMDC intermediate vs favorable 3.39 (0.43–27.02) 0.248

IMDC poor vs favorable 4.82 (0.56–41.45) 0.152

Nephrectomy yes 0.21 (0.11–0.40) < 0.001

High CAR 2.29 (1.23–4.27) 0.009

High SII 2.96 (1.58–5.53) < 0.001

"AI-assisted technologies" should be moved to the end of the manuscript in the section of declarations.

Response: Thank you for the comment. We removed the AI-assisted technologies statement from the Material and methods section and moved it to the Declarations / Additional Information section at the end of the manuscript.

The details of the revision are as follows: The AI-assisted technologies statement has been moved to the end of the manuscript (page 16, lines 475–478).

A result must stay either in text, in a table, or in a figure. Do not duplicate the results

Response: We thank the Academic Editor for this comment. We have revised the Results section, tables, and figures to reduce duplication and improve readability. Detailed numerical results, including hazard ratios and 95% confidence intervals, are presented in the tables where appropriate. The Kaplan–Meier figures were simplified to focus on the survival curves and patient-at-risk tables. In the Results section, we have retained only the key findings needed to guide the reader and have avoided repeating all the numerical results already shown in the tables.

The details of the revision are as follows: The Results section, tables, and figures have been revised to ensure that each result is presented in the most appropriate location and to reduce unnecessary duplication between the text, tables, and figures.

Table 1 should also include univariable analysis for targeted outcomes.

Response: We thank the Academic Editor for this suggestion. We have revised Table 1 to include univariable Cox regression analyses for OS, which was defined as the primary outcome of this study. Specifically, columns showing univariable HRs with 95% CIs and p-values were added to Table 1 for the baseline characteristics and inflammatory/nutritional markers included in the table.

High CAR and SII were both associated with shorter OS in univariable analyses. The HRs were 2.70 for high CAR (95% CI, 1.54–4.74; p < 0.001) and 2.80 for high SII (95% CI, 1.59–4.94; p < 0.001).

The details of the revision are as follows: Table 1 has been revised to include univariable Cox regression results for OS, including HRs with 95% CIs and p-values.

Transparency in multivariable analysis is missing. It is unclear how the multivariable analysis was conducted.

Response: We thank the Academic Editor for this valuable comment. We agree that the original description of the multivariable modeling strategy was insufficiently detailed. Therefore, we revised the Statistical Analysis subsection to explicitly describe the construction of the main multivariable Cox model.

The main multivariable model was defined a priori for OS and included IMDC risk classification, nephrectomy status, high CAR, and high SII. IMDC risk classification and nephrectomy status were selected as clinically relevant prognostic factors, whereas high CAR and SII were included as the main biomarkers of interest. Variables were not selected based on univariable p-values.

The number of death events was 56. Because IMDC risk classification was entered as a three-level categorical variable, the main model included five model parameters corresponding to approximately 11 events per model parameter. Additional covariates were not simultaneously included in the main model to reduce the risk of overfitting. Instead, treatment heterogeneity, histological heterogeneity, and the influence of dichotomized cutoffs were evaluated in sensitivity analyses, including a model additionally adjusted for ICI-based first-line therapy, an analysis restricted to patients with confirmed clear-cell RCC, and models treating CAR and SII as log-transformed standardized continuous variables.

The details of the revision are as follows: The Statistical analysis subsection has been revised to clarify the multivariable modeling strategy, variables included in the main model, and event-per-parameter calculation. The Results section and Supplementary Tables S1–S3 have also been revised to present sensitivity analyses evaluating treatment heterogeneity, histological heterogeneity, and continuous biomarker modeling.

Cox regression without including covariates is misleading. This is a major issue.

Response: We thank the Academic Editor for this important comment. We agree that unadjusted Cox regression alone is insufficient to support the independent prognostic value of CAR and SII. Therefore, we have revised the manuscript to include a main multivariable Cox regression model adjusted for clinically relevant covariates, including IMDC risk classification and nephrectomy status.

In this adjusted model, both high CAR and SII remained independently associated with shorter OS. The adjusted HRs were 2.36 for high CAR (95% CI, 1.30–4.27; p = 0.005) and 2.58 for high SII (95% CI, 1.42–4.69; p = 0.002). These results are presented in the Results section and Table 2. We have also clarified the multivariable modeling strategy in the Statistical analysis subsection.

The details of the revision are as follows: The Statistical analysis subsection, Results section, and Table 2 have been revised to include and describe the adjusted multivariable Cox regression analysis (page 6–7, lines 147–161; page 8–9, lines 216–223).

The increase in C-index (Table 3) is given by considering more variable. Which is the clinical relevance, considering that the values are low?

Response: We thank the Academic Editor for this important comment. We agree that the increase in the C-index should be interpreted cautiously because the expanded model included additional variables and the absolute discriminatory performance remained modest.

In the revised manuscript, we describe the increase in the optimism-corrected C-index from 0.641 for the IMDC model to 0.733 for the IMDC+CAR+SII model as a modest and exploratory improvement in discrimination. We also clarified that this improvement does not by itself establish clinical utility and that CAR and SII should be considered adjunctive markers to complement, rather than replace, established clinical risk models.

The details of the revision are as follows: The Results, Discussion, and Conclusion have been revised to provide a more cautious interpretation of the C-index improvement and to emphasize that these findings require external validation before clinical implementation (page 9, lines 239–243; page 11–12, lines 307–312; page 13, lines 346–359).

AUC should be reported with 95% confidence intervals. As are looking, none AUC show clinical relevance.

Response: We thank the Academic Editor for this important comment. We agree that AUC values should be reported with measures of uncertainty and that the observed AUC values should not be interpreted as definitive evidence of clinical relevance.

We added 95% confidence intervals for the time-dependent AUCs at 1, 3, and 5 years. In the revised analysis, the time-dependent AUCs for the IMDC+CAR+SII model were 0.728 (95% CI, 0.581–0.875), 0.756 (95% CI, 0.648–0.864), and 0.767 (95% CI, 0.630–0.904) at 1, 3, and 5 years, respectively. The corresponding AUCs for the IMDC model were 0.602 (95% CI, 0.479–0.725), 0.672 (95% CI, 0.571–0.773), and 0.606 (95% CI, 0.476–0.735), respectively. We also added 95% confidence intervals for the Brier scores as measures of uncertainty for prediction error.

As the AUC values remained moderate and the confidence intervals were relatively wide, we have revised the Results and Discussion to interpret these findings as exploratory rather than definitive evidence of clinical utility. In addition, we clarified that external validation is required to determine whether the addition of CAR and SII provides clinically meaningful incremental prognostic value.

The details of the revision are as follows: The Results section, Figure 5, and Supplementary Table S4 have been revised to include 95% confidence intervals for the time-dependent AUCs and Brier scores. The interpretation of the model performance analysis has also been revised to avoid overstatement of clinical relevance (page 9–10, lines 244–254; page 13, lines 346–359).

Discussion - Do not make a summary of your study. Start this section by presenting the main conclusion of your study.

Response: We thank the Academic Editor for this valuable comment. We agree that the Discussion should begin with the main conclusion rather than with a descriptive summary of the study design and results. Therefore, we have revised the opening paragraph of the Discussion to first state the main conclusion of the study.

The revised Discussion now begins by stating that pre-treatment CAR and SII are readily available inflammatory biomarkers that may help refine OS risk assessment in patients with mRCC receiving first-line systemic therapy. We also clarified that patients with increased CAR and SII had the poorest survival and that adding these markers to established clinical factors modestly improved the prognostic performance, while emphasizing that external validation is required before clinical implementation.

The details of the revision are as follows: The first paragraph of the Discussion has been revised to present the main conclusion of the study before discussing the supporting results (page 10, lines 264–270).

The clinical relevance of the reported results is not appropriately discussed.

Response: We thank the Academic Editor for this important comment. We agree that the clinical relevance of the reported findings was not discussed sufficiently in the original manuscript. Therefore, we revised the Discussion to clarify how CAR and SII may be interpreted in clinical practice while avoiding the overstatement of their immediate clinical applicability.

In the revised Discussion, we state that CAR and SII may be useful adjunctive markers to complement, rather than replace, the established clinical risk models. As these indices are inexpensive and readily available from routine pretreatment laboratory data, they may help refine baseline risk assessment, support prognostic discussions, identify patients who may require closer follow-up, and serve as stratification or adjustment factors in future clinical trials. We also clarified that the present findings should not be used alone to select systemic therapy or alter treatment decisions.

The details of the revision are as follows: The Discussion has been revised to clarify the potential clinical relevance of CAR and SII as adjunctive markers for baseline risk assessment, prognostic discussion, closer follow-up, and trial stratification, while emphasizing that they should not be used alone to guide treatment decisions (page 11–12, lines 307–312).

The generalizability of the reported results is not appropriately discussed.

Response: We thank the Academic Editor for this valuable comment. We have expanded the limitations section to discuss generalizability more explicitly. The revised Discussion now states that the generalizability of the findings should be interpreted in light of the study setting.

Specifically, we clarified that this was a single-center retrospective study conducted in Japan and that the cohort included patients treated across different therapeutic eras. In addition, only 24 patients received ICI-based first-line therapy, whereas most patients received TKI-based therapy. Therefore, the findings may not be directly generalizable to contemporary cohorts predominantly treated with current ICI-based combination regimens or to different healthcare systems, treatment patterns, and patient populations.

The details of the revision are as follows: The limitations section of the Discussion has been revised to address generalizability more explicitly and to emphasize the need for external validation in larger contemporary multicenter cohorts (page 12, lines 328–332; page 13, lines 358–359).

The corrected parts in the text of the revised manuscript are shown in red.

In the revised version, Dr. Nobuhito Muramoto has been added as a co-corresponding author. This change reflects his substantial contributions to the revision of the manuscript, including additional analyses, interpretation of results, manuscript revision, and the preparation of responses to the Academic Editor and Reviewers. All authors reviewed and approved this change.

We hope that the revised manuscript has been satisfactorily improved and has achieved priority and quality appropriate for publication in PLOS ONE.

Reply to Reviewer #1

We thank Reviewer #1 for their careful review and constructive comments. We have revised the manuscript and performed additional analyses in response to these comments, as detailed below.

Comments from Reviewer #1:

This retrospective single-center study investigates the prognostic value of two inflammation-based biomarkers—C-reactive protein-to-albumin ratio (CAR) and systemic immune-inflammation index (SII)—in patients with metastatic renal cell carcinoma (mRCC). 117 patients treated with first-line systemic therapy between 2001 and 2022 were included. The authors evaluated pretreatment CAR and SII using time-dependent ROC analyses to define optimal cutoffs (CAR: 0.0731; SII: 533.81), followed by Kaplan–Meier survival analyses and Cox proportional hazards models adjusted for IMDC risk classification and nephrectomy status. The authors conclude that CAR and SII are inexpensive, readily available biomarkers that may enhance prognostic stratification beyond established IMDC criteria, though external validation is required.

MAJOR COMMENTS:

Comment 1: Clarify treatment heterogeneity and adjust if possible. Provide stratified analyses (e.g., TKI vs ICI era), or alternatively, include treatment type in multivariable models or sensitivity analyses.

Response: We thank the Reviewer for this important comment. We agree that treatment heterogeneity is an important issue in this retrospective cohort, which included patients treated across different therapeutic eras. We reviewed the description of first-line treatment and corrected an inconsistency in the text so that the number of patients receiving ICI-based first-line therapy is consistent with Table 1 and the analyses.

To address treatment heterogeneity, we performed an additional sensitivity analysis that included ICI-based first-line therapy as a covariate in the multivariable Cox proportional hazards model. This model included IMDC risk classification, nephrectomy status, high CAR, high SII, and ICI-based first-line therapy. After this additional adjustment, high CAR and high SII remained significantly associated with shorter OS. The adjusted HRs were 2.36 for high CAR (95% CI, 1.31–4.27; p = 0.004) and 2.50 for high SII (95% CI, 1.33–4.70; p = 0.004). ICI-based first-line therapy itself was not significantly associated with OS in this model (adjusted HR, 0.86; 95% CI, 0.32–2.33; p = 0.767). These results have been presented in Supplementary Table S1.

As only 24 patients received ICI-based first-line therapy, formal stratified analyses by treatment type were considered exploratory and potentially underpowered. Therefore, we addressed treatment heterogeneity primarily through covariate adjustment and have added this limitation to the Discussion. We also harmonized the coding and reference categories used in the Cox regression tables to improve consistency.

The details of the revision are as follows: The Results section, Supplementary Table S1, and Discussion have been revised to clarify treatment heterogeneity, correct the inconsistency in the number of patients receiving ICI-based first-line therapy, report the sensitivity analysis adjusted for ICI-based first-line therapy, and harmonize the presentation of Cox regression results (page 7, lines 185–187; page 9, lines 224–229; page 12, lines 328–332).

Comment 2: Justify and contextualize cutoff values. Emphasize that cutoffs are exploratory, compare quantitatively with prior literature, and consider reporting continuous-variable analyses alongside dichotomized ones.

Response: We thank the Reviewer for this valuable comment. We agree that the cutoff values should be justified and interpreted cautiously. We revised the Methods and Discussion to clarify that the CAR and SII cutoffs were derived in the present cohort using time-dependent ROC analysis at a prespecified 5-year time horizon. We also emphasized that these cutoffs are exploratory and should not be considered universally applicable thresholds.

We further added context by comparing our cutoff values with those reported in previous studies. The CAR cutoff in the present study was 0.0731, close to the cutoff of 0.08 reported in a previous study of patients with RCC following nephrectomy. The SII cutoff in the present study was 533.81, broadly comparable to previously reported SII cutoffs, such as 628 in patients with localized RCC and 730 in patients with metastatic RCC. However, cutoff values vary across studies because of differences in patient populations, treatment settings, laboratory conventions, time horizons, and statistical methods.

To address the concern regarding dichotomization, we additionally analyzed CAR and SII as log-transformed standardized continuous variables. In the sensitivity analysis, both markers remained significantly associated with OS. The adjusted HRs per 1 standard deviation increase were 1.58 for log-transformed CAR (95% CI, 1.09–2.28; p = 0.015) and 1.97 for log-transformed SII (95% CI, 1.30–2.98; p = 0.001). These results have been added to Supplementary Table S3.

The details of the revision are as follows: The Material and methods, Results, Discussion, and Supplementary Table S3 have been revised to justify and contextualize the cutoff values, emphasize their exploratory nature, and report continuous-variable analyses in addition to the dichotomized analyses (page 5–6, lines 129–138; page 7, lines 165–171; page 9, lines 235–238; page 13, lines 352–356).

Comment 3: Strengthen multivariable modeling by providing a rationale for excluding key variables and considering adding at least performance status (continuous or categorical) and histology (clear vs non-clear). Report the number of events per variable (EPV).

Response: We thank the Reviewer for this important comment. We agree that the multivariable modeling strategy required clearer justification. The number of death events was 56. To reduce the risk of overfitting, we used a parsimonious main multivariable model including IMDC risk classification, nephrectomy status, high CAR, and high SII. This model included five parameters because IMDC risk classification was entered as a three-level categorical variable, corresponding to approximately 11 events per model parameter.

We considered adding performance status and histology to the main model. However, the performance status was highly imbalanced, with only two patients having KPS <80. Histology was not included in the main model because histological subtype was unknown in 23 patients. Therefore, directly adding these variables to the primary model could have reduced model stability and interpretability.

Instead, we addressed histological heterogeneity using a sensitivity analysis restricted to patients with confirmed clear cell RCC. In this analysis, high SII remained significantly associated with shorter OS (adjusted HR, 3.39; 95% CI, 1.44–8.00; p = 0.005), whereas high CAR showed an association in the same direction but did not reach statistical significance (adjusted HR, 1.95; 95% CI, 0.91–4.21; p = 0.087). These results have been added to Supplementary Table S2.

The details of the revision are as follows: The Statistical analysis subsection has been revised to describe the modeling rationale and events-per-parameter calculation, including the rationale for not adding performance status or histology to the main model (page 6–7, lines 150–160). Additionally, the Results section and Supplementary Table S2 have been revised to present the clear cell RCC-only sensitivity analysis, and the Discussion has been revised to acknowledge the limitations related to histological heterogeneity (page 9, lines 230–234; page 13, lines 342–346).

Comment 4: Although discrimination (C-index) and Brier score are reported, please provide calibration assessment and include calibration plots or at least calibration slope/CITL.

Response: We thank the Reviewer for this important suggestion. We agree that discrimination metrics alone are insufficient for evaluating prognostic model performance. Therefore, we added a calibration assessment to the revised analysis.

Specifically, we assessed the calibration of the IMDC and IMDC+CAR+SII models at 1, 3, and 5 years using calibration plots based on predicted and observed survival probabilities. The calibration plots showed generally acceptable agreement between predicted and observed survival probabilities for both models, although some deviations were observed, particularly at later time points. The calibration slopes were also calculated using the Cox linear predictor to provide a quantitative summary of the calibration. The calibration slopes were 1.000 (95% CI, 0.363–1.637) for the IMDC model and 1.000 (95% CI, 0.632–1.368) for the IMDC+CAR+SII model. These results were added to the Results section, Supplementary Figure S3, and Supplementary Table S5.

We have also revised the Discussion to state that the calibration assessment should be interpreted cautiously because of the modest sample size, single-center retrospective design, and limited number of patients at risk at later time points.

The details of the revision are as follows: The Results section, Discussion, Supplementary Figure S3, and Supplementary Table S5 have been revised to include calibration assessment and provide a cautious interpretation of model calibration (page 10, lines 255–260; page 13, lines 346–351).

Comment 5: A ΔC-index of 0.092 is notable but requires contextual interpretation of whether it is clinically meaningful or not. Compare to thresholds used in prognostic model evaluation.

Response: We thank the reviewer for this valuable comment. We agree that the observed ΔC-index should be interpreted in a clinical context and not be regarded as sufficient evidence of clinical usefulness by itself.

In the revised manuscript, we describe the increase in the optimism-corrected C-index from 0.641 for the IMDC model to 0.733 for the IMDC+CAR+SII model as a modest improvement. Although the absolute increase of 0.092 suggests improved discrimination, there is no universally accepted ΔC-index threshold that defines clinically meaningful improvement across prognostic models, and the interpretation depends on the clinical setting, baseline model performance, outcome, and intended use of the model. Therefore, we avoided claiming that the observed ΔC-index alone establishes clinical utility.

To provide a broader context, we also evaluated model performance using time-dependent AUCs, Brier scores, calibration plots, calibration slopes, and decision curve analysis. These analyses suggested that adding CAR and SII to IMDC may improve discrimination and slightly reduce prediction error, with generally acceptable calibration and a higher net benefit at 1 and 3 years. However, because the expanded model included additional variables and the absolute predictive performance remained limited, we have revised the Results and Discussion to describe the findings as exploratory and emphasize the need for external validation.

The details of the revision are as follows: The Results and Discussion have been revised to provide a more cautious interpretation of the C-index improvement and emphasize that external validation is required before clinical implementation (page 9, lines 239–244; page 13, lines 346–359).

Comment 6: Expand on clinical applicability: How would CAR/SII change decision-making? Risk-adapted therapy? Surveillance? Trial stratification?

Response: We thank the Reviewer for this helpful suggestion. We agree that the potential clinical applicability of CAR and SII should be more explicitly discussed. Therefore, we expanded the Discussion to clarify the potential application of these markers in clinical and research settings.

Because CAR and SII are calculated from routine pretreatment laboratory data, they may serve as accessible adjuncts to established risk stratification systems, such as the IMDC risk classification. In practical terms, elevated CAR and SII may help identify patients who could benefit from closer follow-up, more careful prognostic discussions, and consideration of risk-adapted surveillance strategies. In addition, these markers may be useful for stratification or adjustment in future clinical trials evaluating systemic therapies for metastatic RCC.

In addition, we revised the Discussion to avoid overstating clinical applicability. We now clearly state that CAR and SII should not be used alone to select systemic therapy or alter clinical management based on the present retrospective single-center study. External validation in larger contemporary cohorts, particularly in patients treated with current ICI-based combination therapies, is required prior to clinical implementation.

The details of the revision are as follows: The Discussion has been expanded to clarify the potential clinical applicability of CAR and SII, including their possible use for baseline risk assessment, prognostic discussion, closer follow-up, and trial stratification, while emphasizing that they should not be used alone to guide treatment decisions without external validation (page 11–12, lines 307–312).

Comment 7: Provide a clearer mechanism for data access. Consider anonymized dataset sharing if possible.

Response: We thank the Reviewer for this comment. We revised the Data availability statement to clarify that the data cannot be made publicly available because it contains potentially identifiable clinical information from a single institution. We have also added a clear mechanism for data access requests. Data may be available to qualified researchers upon reasonable request and with the approval of the Ethics Committee of Juntendo University, referencing the approval number E23-0275-H01.

The details of the revision are as follows: The Data availability statement has been revised to state that data access requests should be directed to the Ethics Committee of Juntendo University, Tokyo, Japan, at hongo-rinri@juntendo.ac.jp, referencing the approval number E23-0275-H01 (page 16, lines 464–470).

Minor COMMENTS:

Comment 1: The language is generally clear, but minor grammatical inconsistencies persist (likely from mixed editing sources). There is some redundancy in the Introduction and Discussion.

Response: We thank the Reviewer for this helpful comment. We carefully reviewed the manuscript and revised the language to improve grammar, consistency, and readability. We also shortened or removed redundant descriptions in the Introduction and Discussion. In addition, the revised manuscript has been reviewed by a professional English editing service to further improve language consistency.

Comment 2: In Figure 1 (page 27), multiple ROC curves are overlaid. Consider simplifying or separating panels for readability.

Response: We agree that the original Figure 1 was visually dense because multiple time-dependent ROC curves were overlaid. To improve readability, we have revised Figure 1 by reducing the number of displayed ROC curves from five time points to three clinically relevant time points. The revised Figure 1 now shows the time-dependent ROC curves for CAR and SII at 1, 3, and 5 years.

The details of the revision are as follows: Figure 1 has been revised to improve readability by reducing the number of overlaid ROC curves from 1, 2, 3, 4, and 5 years to 1, 3, and 5 years.

Comment 3: In Figure 2 (page 28), median survival annotations are helpful but visually dense.

Response: We thank the Reviewer for this helpful comment. We have revised Figure 2 to improve readability by removing the median survival annotations from the plot area and avoiding additional numerical annotations overlaid on the Kaplan–Meier curves.

The details of the revision are as follows: Figure 2 and its legend have been revised accordingly.

Comment 4: Use consistent formatting for CAR and SII (e.g., “high vs low” vs “≥ cutoff”).

Response: We agree with your comment. We have standardized the terminology in the manuscript. We now use “high CAR,” “low CAR,” “high SII,” and “low SII” consistently in the text, tables, and figure legends. These categories are defined as CAR ≥ 0.0731, CAR < 0.0731, SII ≥ 533.81, and SII < 533.81, respectively.

The details of the revision are as follows: The manuscript, tables, and figure legends have been revised to ensure consistent terminology for CAR and SII categories.

Comment 5: Please include number at risk tables (already present, but ensure clarity) and exact p-values instead of thresholds where possible.

Response: We thank the Reviewer for this comment. We confirmed that the Kaplan–Meier figures included number-at-risk tables and have revised them to improve clarity. To avoid visual crowding, unnecessary numerical annotations were removed from the plotting area. Exact p-values are reported in the Results section where appropriate. For very small p-values, we retained the conventional notation of p < 0.001.

The details of the revision are as follows: The Kaplan–Meier figures and legends have been revised to improve readability, and the Results section has been revised to report p-values where appropriate.

Comment 6: IMDC appears non-significant in multivariable analysis. This is unexpected and should be discussed explicitly.

Response: We thank the Reviewer for this valuable comment. We agree that the non-significance of IMDC risk classification in the multivariable model was unexpected and requires explicit discussion.

We have added a statement to the Discussion noting that IMDC risk classification was not statistically significant in the main multivariable model. We also clarified that this finding should not be interpreted as evidence that IMDC lacks prognostic value. Rather, it may be partly explained by the small number of patients in the favorable-risk group, the modest number of death events, the potential overlap between systemic inflammatory markers and IMDC components, and the inclusion of nephrectomy status and CAR/SII in the same model. Therefore, we interpreted this finding as reflecting the limitations of model stability and cohort-specific factors in this single-center retrospective cohort.

The details of the revision are as follows: The Discussion has been revised to explicitly address the non-significance of IMDC risk classification in the multivariable model (page 12–13, lines 339–341).

The corrected parts in the text of the revised manuscript are shown in red.

In the revised version, Dr. Nobuhito Muramoto has been added as a co-corresponding author. This change reflects his substantial contributions to the revision of the manuscript, including additional analyses, interpretation of results, manuscript revision, and the preparation of responses to the Academic Editor and Reviewers. All authors have reviewed and approved this change.

We hope that the revised manuscript has been satisfactorily improved and achieves the priority and quality appropriate for publication in PLOS ONE.

Reply to Reviewer #2

We thank Reviewer #2 for their careful review and constructive comments. We have revised the manuscript and performed additional analyses in response to these comments, as detailed below.

Comments from Reviewer #2:

Thank you to the authors for submitting this manuscript describing a study in which clinical outcomes of mRCC over 5yrs in a single centre were studied in the context of two inflammatory indices (CAR & SII). I have minor queries:

Comment 1: There are very occasional minor, unusual turns of phrase for correction.

Response: We thank the Reviewer for this comment. We have carefully reviewed the manuscript and revised the text to correct unusual phrasing and improve clarity, consistency, and readability. In addition, the revised manuscript has been reviewed by a professional English language editing service.

Comment 2: Fig 1 - the performance of CAR degrades somewhat over time compared to SII, which is well preserved. Can the authors explore this?

Response: We thank the Reviewer for this insightful observation. We agree that the time-dependent ROC curves suggest that the discriminatory performance of CAR may decrease over time compared with that of the SII. To explore this observation, we quantitatively compared the time-dependent AUCs for CAR and SII at 1, 3, and 5 years.

The AUC for CAR decreased from 0.798 (95% CI, 0.707–0.890) at 1 year to 0.656 (95% CI, 0.505–0.807) at 5 years. In contrast, the AUC for SII was relatively preserved, from 0.622 (95% CI, 0.468–0.777) at 1 year to 0.754 (95% CI, 0.614–0.895) at 5 years.

These findings are consistent with the Reviewer’s observation and suggest that CAR may show stronger short-term discrimination, whereas SII may provide more sustained discrimination over longer follow-up periods. This pattern may reflect differences in the biological information captured by CAR and SII; however, because this was an exploratory assessment based only on pretreatment biomarkers and the confidence intervals were relatively wide, the results should be interpreted cautiously.

Comment 3: Are the authors able to explore in practical terms the impacts on outcomes with either standard deviations (or deciles/quartiles) of the variance of the CAR & SII?

Response: We thank the Reviewer for this important suggestion. We agree that evaluating CAR and SII only as dichotomized variables may not fully capture their continuous relationship with outcomes. To address this point and reduce reliance on dichotomized cutoff values, we additionally analyzed CAR and SII as log-transformed standardized continuous variables.

In this sensitivity analysis, both markers remained significantly associated with OS. The adjusted HRs per 1 standard deviation increase were 1.58 for log-transformed CAR (95% CI, 1.09–2.28; p = 0.015) and 1.97 for log-transformed SII (95% CI, 1.30–2.98; p = 0.001). We used the standard deviation-based approach because it preserves the continuous nature of the biomarkers and avoids the introduction of additional arbitrary categories, such as quartiles or deciles, in this modest-sized cohort. These results have been added to Supplementary Table S3.

The details of the revision are as follows: The Statistical analysis subsection and Results section have been revised, and Supplementary Table S3 has been added to show the sensitivity analysis treating CAR and SII as log-transformed standardized continuous variables (page 7, lines 169–171; page 9, lines 235–238).

Comment 4: Whilst this is further observational supportive evidence for a role of inflammation in RCC cancerogenesis, this remains a somewhat vague & impersonal association only identified in retrospect and without a well-identified cause of inflammation. Are there any future therapeutic or preventative approaches that the authors might suggest exploring?

Response: We thank the Reviewer for this important comment. We agree that the present study provides observational and retrospective evidence only and that CAR and SII should be interpreted as host inflammatory markers rather than as mechanistic evidence identifying a specific cause of inflammation. We have revised the Discussion to clarify this limitation and describe future research directions.

In addition, we noted that future prospective studies should evaluate whether longitudinal changes in CAR and SII reflect treatment response, tumor progression, infection, comorbidities, nutritional deterioration, sarcopenia, or other potentially modifiable inflammatory states. Interventions targeting modifiable contributors to systemic inflammation, such as nutritional optimization, management of infection or comorbid inflammatory conditions, improvement of body composition, and supportive care during systemic therapy, may represent reasonable areas for future investigation. However, we avoided suggesting that CAR or SII currently support any specific anti-inflammatory or preventive intervention.

The details of the revision are as follows: The Discussion has been revised to clarify the observational nature of the findings and describe future research directions, including the prospective evaluation of longitudinal inflammatory markers and potentially modifiable contributors to systemic inflammation (page 12, lines 313–321).

The corrected parts in the text of the revised manuscript are shown in red.

In the revised version, Dr. Nobuhito Muramoto has been added as a co-corresponding author. This change reflects his substantial contributions to the revision of the manuscript, including additional analyses, interpretation of results, manuscript revision, and the preparation of responses to the Academic Editor and Reviewers. All authors have reviewed and approved this change.

We hope that the revised manuscript has been satisfactorily improved and achieves the priority and quality appropriate for publication in PLOS ONE.

Reply to Reviewer #3

We thank Reviewer #3 for their careful review and constructive comments. We have revised the manuscript and performed additional analyses in response to these comments, as detailed below.

Comments from Reviewer #3:

Congratulations to the authors on this interesting study. However, I have two concerns regarding the presented results.

Comment 1: In the Results section, the authors report that 81 patients underwent nephrectomy. Could the authors clarify why the remaining patients in the cohort did not undergo nephrectomy? This information was not clearly presented in the manuscript and may be important for understanding patient selection and potential sources of bias.

Response: We thank the Reviewer for this important comment. In this retrospective cohort study, 81 patients underwent nephrectomy, whereas 36 patients did not. The reasons for not undergoing nephrectomy were not uniform and included advanced metastatic burden, limited suitability for surgery, treatment strategies prioritizing systemic therapy, and lack of indication for cytoreductive nephrectomy at the discretion of the treating physician.

The details of the revision are as follows: The Discussion has been revised to clarify that nephrectomy status should be interpreted cautiously because the absence of nephrectomy may reflect patient selection and disease severity and residual confounding and selection bias related to nephrectomy status cannot be excluded (page 12, lines 334–339).

Comment 2: According to the Results table, 15 patients had a non–clear cell histological subtype and 23 patients had an unknown histological subtype. Nevertheless, these patients were included in the analyses. Could the authors clarify how the CAR and SII analyses were performed and interpreted for these patients, given that they did not have confirmed metastatic clear cell renal cell carcinoma (mRCC)?

Response: We thank the Reviewer for this valuable comment. We agree that the inclusion of patients with non-clear cell RCC or unknown histology may affect the interpretation of the CAR and SII analyses.

The main analysis included all patients who were managed for metastatic RCC and received first-line systemic therapy at our institution. This approach was chosen because this study aimed to evaluate pretreatment inflammatory biomarkers in a real-world metastatic RCC treatment cohort rather than in a cohort limited to patients with confirmed clear cell RCC. CAR and SII were calculated from pretreatment peripheral blood data in the same manner for all patients, regardless of histological subtype.

To address the Reviewer’s concern regarding histological heterogeneity, we performed a sensitivity analysis restricted to patients with confirmed clear cell RCC. In this analysis, high SII remained significantly associated with shorter OS (adjusted HR, 3.39; 95% CI, 1.44–8.00; p = 0.005). The association for high CAR was in the same direction but did not reach statistical significance (adjusted HR, 1.95; 95% CI, 0.91–4.21; p = 0.087), likely reflecting the reduced sample size and number of events. These results have been added to Supplementary Table S2.

The details of the revision are as follows: The Statistical analysis subsection has been revised to clarify that histological subtype was not included in the primary model because it was unknown in 23 patients and that histological heterogeneity was assessed in a sensitivity analysis restricted to patients with confirmed clear cell RCC. The Results section and Supplementary Table S2 have been revised to present the clear cell RCC-only sensitivity analysis, and the Discussion has been revised to acknowledge histological heterogeneity and the inclusion of patients with unknown histology as limitations (page 6, lines 156–158; page 9, lines 230–234; page 13, lines 342–346).

The corrected parts in the text of the revised manuscript are shown in red.

In the revised version, Dr. Nobuhito Muramoto has been added as a co-corresponding author. This change reflects his substantial contributions to the revision of the manuscript, including additional analyses, interpretation of results, manuscript revision, and the preparation of responses to the Academic Editor and Reviewers. All authors have reviewed and approved this change.

We hope that the revised manuscript has been satisfactorily improved and achieves the priority and quality appropriate for publication in PLOS ONE.

Reply to Reviewer #4

We thank Reviewer #4 for their careful review and constructive comments. We have revised the manuscript and performed additional analyses in response to these comments, as detailed below.

Comments from Reviewer #4:

The term outcomes has been used a lot throughout the manuscript. It has not been clearly defined what outcome exactly is/are?

Especially the starting portion of the discussion- mentiones about clinical outcomes which seems a vague term in the contect of the discussion.

Response: We thank the Reviewer for this important comment. We agree that the term “outcomes” was used too broadly in the original manuscript and could be interpreted as vague. We revised the manuscript to more clearly define the study outcomes. Specifically, OS is now defined as the primary outcome and PFS as the secondary outcome in the Abstract and Statistical analysis subsection. Additionally, we revised vague uses of “clinical outcomes” and “outcomes” in the Results and Discussion to more specific terms, such as “OS,” “PFS,” “PFS and OS,” or “survival outcomes,” depending on the context.

In particular, the opening paragraph of the Discussion has been revised to focus on the main findings for OS. The revised Discussion now states that pretreatment CAR and SII were independently associated with OS in patients with mRCC receiving first-line systemic therapy and that their combined use may provide additional prognostic information beyond established clinical factors.

The details of the revision are as follows: The Abstract and Statistical analysis subsection have been revised to define OS as the primary outcome and PFS as the secondary outcome. The Results and Discussion have been revised to replace vague uses of “clinical outcomes” and “outcomes” with more specific outcome terminology where appropriate (page 2, lines 34–35; page 6, lines 140–141; page 10, lines 264–271).

The corrected parts in the text of the revised manuscript are shown in red.

In the revised version, Dr. Nobuhito Muramoto has been added as a co-corresponding author. This change reflects his substantial contributions to the revision of the manuscript, including additional analyses, interpretation of results, manuscript revision, and the preparation of responses to the Academic Editor and Reviewers. All authors have reviewed and approved this change.

We hope that the revised manuscript has been satisfactorily improved and achieves the priority and quality appropriate for publication in PLOS ONE.

Attachment Submitted filename: 260806_1_Revise_comment.docx

# Peer review: Decision Letter 1

**DOI:** 10.1371/journal.pone.0345724.r003

**Authors:** Sorana Bolboacă

16 Sep 2026

<p>Clinical utility of combining the C-reactive protein-to-albumin ratio and systemic immune-inflammation index for prognostic assessment in metastatic renal cell carcinoma

PONE-D-26-11056R1

Dear Dr. Nagata,

We’re pleased to inform you that your manuscript has been judged scientifically suitable for publication and will be formally accepted for publication once it meets all outstanding technical requirements.

Within one week, you’ll receive an e-mail detailing the required amendments. When these have been addressed, you’ll receive a formal acceptance letter and your manuscript will be scheduled for publication.

An invoice will be generated when your article is formally accepted. Please note, if your institution has a publishing partnership with PLOS and your article meets the relevant criteria, all or part of your publication costs will be covered. Please make sure your user information is up-to-date by logging into Editorial Manager at Editorial Manager® and clicking the ‘Update My Information' link at the top of the page. For questions related to billing, please contact billing support.

If your institution or institutions have a press office, please notify them about your upcoming paper to help maximize its impact. If they’ll be preparing press materials, please inform our press team as soon as possible -- no later than 48 hours after receiving the formal acceptance. Your manuscript will remain under strict press embargo until 2 pm Eastern Time on the date of publication. For more information, please contact onepress@plos.org.

Kind regards,

Sorana D. Bolboacă, Ph.D., M.Sc., M.D.

Academic Editor

PLOS One

Reviewers' comments:

Reviewer's Responses to Questions

**Comments to the Author**

Reviewer #2: All comments have been addressed

Reviewer #4: All comments have been addressed

**********

2. Is the manuscript technically sound, and do the data support the conclusions?

Reviewer #2: Yes

Reviewer #4: Yes

**********

3. Has the statistical analysis been performed appropriately and rigorously?

Reviewer #2: I Don't Know

Reviewer #4: Yes

**********

4. Have the authors made all data underlying the findings in their manuscript fully available?

The PLOS Data policy

Reviewer #2: Yes

Reviewer #4: Yes

**********

5. Is the manuscript presented in an intelligible fashion and written in standard English?

Reviewer #2: Yes

Reviewer #4: Yes

**********

Reviewer #2: Thank you to the authors for submitting this revised manuscript describing a study in which clinical outcomes of mRCC over 5yrs in a single centre were studied in the context of two inflammatory

indices (CAR & SII). My previous queries have been addressed and I do not have additional ones at this time.

Reviewer #4: The previous comments and suggestions have been extensive and a reliable valid effort had been made by the authors in answering all revisions.

**********

what does this mean?). If published, this will include your full peer review and any attached files.

If you choose “no”, your identity will remain anonymous but your review may still be made public.

**Do you want your identity to be public for this peer review?**  For information about this choice, including consent withdrawal, please see our Privacy Policy

Reviewer #2: No

Reviewer #4: **Yes:**  Hari Naga Garapati

**********

# Editor report: Acceptance letter

**DOI:** 10.1371/journal.pone.0345724.r004

**Authors:** Sorana Bolboacă

PONE-D-26-11056R1

PLOS One

Dear Dr. Nagata,

I'm pleased to inform you that your manuscript has been deemed suitable for publication in PLOS One. Congratulations! Your manuscript is now being handed over to our production team.

At this stage, our production department will prepare your paper for publication. This includes ensuring the following:

* All references, tables, and figures are properly cited

* All relevant supporting information is included in the manuscript submission,

* There are no issues that prevent the paper from being properly typeset

You will receive further instructions from the production team, including instructions on how to review your proof when it is ready. Please keep in mind that we are working through a large volume of accepted articles, so please give us a few days to review your paper and let you know the next and final steps.

Lastly, if your institution or institutions have a press office, please let them know about your upcoming paper now to help maximize its impact. If they'll be preparing press materials, please inform our press team within the next 48 hours. Your manuscript will remain under strict press embargo until 2 pm Eastern Time on the date of publication. For more information, please contact onepress@plos.org.

You will receive an invoice from PLOS for your publication fee after your manuscript has reached the completed accept phase. If you receive an email requesting payment before acceptance or for any other service, this may be a phishing scheme. Learn how to identify phishing emails and protect your accounts at https://explore.plos.org/phishing.

If we can help with anything else, please email us at customercare@plos.org.

Thank you for submitting your work to PLOS One and supporting open access.

Kind regards,

PLOS One Editorial Office Staff

on behalf of

Professor Sorana D. Bolboacă

Academic Editor

PLOS One
